# Haplotype-resolved genome assembly of the colored calla lily (*Zantedeschia elliottiana*) provides new insights into the evolution of the family Araceae

**DOI:** 10.1186/s43897-025-00192-w

**Published:** 2026-02-09

**Authors:** Yi Wang, Tuo Yang, Di Wang, Rongxin Gou, Yin Jiang, Zhen Zeng, Guojun Zhang, Yuhong Zheng, Dan Gao, Liyang Chen, Xiuhai Zhang, Nan Ma, Zunzheng Wei

**Affiliations:** 1 Institute of Grassland, Flowers and Ecology, Beijing Academy of Agriculture and Forestry Sciences, Beijing, 100097 China; 2 Beijing Key Laboratory of Development and Quality Control of Ornamental Crops, Department of Ornamental Horticulture, College of Horticulture, China Agricultural University, Beijing, China; 3 College of Horticultural Science and Technology, Hebei Key Laboratory of Horticultural Germplasm Excavation and Innovative Utilization/Hebei Higher Institute Application Technology Research and Development Center of Horticultural Plant Biological Breeding, Hebei Normal University of Science and Technology, Qinhuangdao, 66004 China; 4 Institute of Botany, Jiangsu Province and Chinese Academy of Sciences, Nanjing Botanical Garden, Mem. Sun Yat-Sen, Nanjing, 210014 Jiangsu China; 5 Smartgenomics Technology Institute, Tianjin, 301700 China

**Keywords:** *Zantedeschia elliottiana*, Transposon, Lignin biosynthesis, Cellulose biosynthesis, Starch synthesis and transport, MIKC^c^ genes

## Abstract

The colored calla lily, a member of the genus *Zantedeschia* in the Araceae family native to South Africa, is a valuable ornamental plant. However, the lack of a high-quality genome has hindered genetic analysis and the identification of key trait-related genes. We successfully assembled a haplotype-resolved genome of the colored calla lily, revealing extensive heterozygosity between homologous chromosomes. Comparative genomics analyses demonstrated that transposon insertions have led to significant differences in genome size among Araceae species. Notably, Araceae species experienced two closely-spaced whole-genome duplication (WGD) events before species divergence. Further analysis revealed that in terrestrial True Araceae species, genes associated with lignin synthase, cellulose synthase, expansin, and sugar transport proteins have undergone expansion, likely contributing to environmental adaptation and tuber starch accumulation. Additionally, we identified a key MIKC^C^ gene that may play a crucial role in spathe development. This study not only elucidates the evolutionary history of Araceae species but also provides valuable data to support functional genomics research and breeding efforts for the colored calla lily.

**Supplementary Information:**

The online version contains supplementary material available at 10.1186/s43897-025-00192-w.

## Core

We present a haplotype-resolved genome of the colored calla lily, uncovering syntenic regions, inversions, translocations, and duplications between the two haplotypes. The analysis highlights the impact of various transposons on genome size within Araceae species. Comparative genomics and synteny analysis provide insights into divergence times and chromosomal rearrangements among Araceae species, revealing a notable expansion of genes related to lignin synthase, cellulose synthase, expansin, and sugar transport proteins in terrestrial True Araceae species. Furthermore, we identified the MIKC^C^ gene associated with spathe development in the colored calla lily. These findings shed light on the genome evolution of Araceae species and pave the way for further research on molecular breeding and key gene functions in colored calla lily.

## Gene and accession numbers

All the raw sequencing data generated during this study have been deposited at the NCBI under BioProject under accessions PRJNA1060881, PRJNA956997, PRJNA957064 and PRJNA1226726. The genome assemblies and annotation files are available at the website 10.6084/m9.figshare.24941703.

## Introduction

The Araceae family, which includes 144 genera and 3,645 species, is an ancient monocotyledonous lineage famous for its diverse morphology, including some of the smallest known angiosperms and some of the tallest stalks and reproductive structures among all angiosperms (Croat 2019; Croat and Ortiz 2020). Araceae species inhabit a wide range of ecosystems, from aquatic (submerged, emergent, free-floating) to epiphytic, climbing, and terrestrial environments. Previous research has indicated that Araceae family’s origins in aquatic or wetland environments evolved with increased aquatic diversity and a shift toward terrestrial habitation (Nauheimer et al. 2012).

Recent studies have divided Araceae into three main groups with eight subfamilies: the original Araceae (Gymnostachydoideae, Orontioideae), the lemnoids (Lemnoideae), and the True Araceae (Lasioideae, Pothoideae, Monsteroideae, Zamioculcadoideae, Aroideae). Proto-Araceae and lemnoids, which form a minor fraction of the Araceae family (6%) and grow primarily in swamps, contrast with the predominantly terrestrial True Araceae (Cusimano et al. 2012; Henriquez et al. 2014). Recent genomic studies of Araceae members, including *Amorphophallus konjac* (Gao et al. 2022), *Colocasia esculenta* (Yin et al. 2020), *Pistia stratiotes* (Qian et al. 2022b), and *Pinellia pedatisecta* (Qian et al. 2022a), have provided valuable phylogenetic insights. However, the genome of the genus *Zantedeschia* remains incomplete, leaving key questions about its evolutionary pathways and metabolic molecular mechanisms unresolved.

*Zantedeschia* spp., commonly known as calla lily, is a perennial herbaceous plant belonging to the Araceae family and is typically found in the swamps and hills of South Africa (Yao et al. 1994). Renowned for its distinctive spathes and ornamental foliage, the calla lily, which can be found in both white and colored varieties, is a globally popular tuberous flowering plant (Chandel et al. 2023). Colored calla lily is an economically significant horticultural crop, leading New Zealand’s cut flower and tuber exports for over three decades, and substantially contributing to the horticultural export revenues of the Netherlands and the United States. Additionally, the leaves of calla lily are traditionally used as a remedy for treating wounds, boils, minor burns, insect stings, and ulcers, highlighting its high economic value and potential for further development.

Whole-genome duplication (WGD) is considered an important evolutionary force and has also played a crucial role in helping plants adapt to changing environmental conditions (Hollister 2014; Jiao et al. 2014; Tang et al. 2009). Ancient WGDs around the Cretaceous-Paleogene boundary, approximately 66 million years ago (MYA), are believed to have allowed angiosperms to thrive in cooler and darker conditions (Vanneste et al. 2014), while WGDs in Caryophyllales have been linked to increased survival during climate shifts in the Paleocene-Eocene (~ 56–54 MYA) and the late Miocene (~ 7 MYA). In the Pooideae subfamily, WGDs are associated with adaptation to lower temperatures and greater species diversification from the mid-Eocene (~ 46 MYA) to the late Oligocene (~ 27 MYA) (Cai et al. 2018; Sessa 2019). Additionally, in the Pooideae subfamily, these WGDs were associated with adaptation to lower temperatures and greater species diversification from the mid-Eocene (~ 46 MYA) to the late Oligocene (~ 27 MYA). Araceae species experienced two closely-spaced WGD events, which are shared among Araceae species and are named α^SP^ and β^SP^, respectively (Wang et al. 2014). These WGD events may be one of the reasons that affect the genome size of Araceae species. Moreover, the insertion of transposons can also directly affect the size of the genome. For instance, long-terminal repeat retrotransposons (LTR-RTs) constitute about 85% of the maize genome, with their insertions being a major factor in its genome size expansion (Schnable et al. 2009). Similarly, transposon insertions contribute to the variation in genome size observed in Araceae species (Gao et al. 2022).

Within the Araceae family, there are both aquatic and terrestrial species. They face completely different living environments and have evolutionary differences, which lead to their differences in morphogenesis and plant architecture. Due to the need to cope with harsher environmental challenges, such as prolonged drought and high-intensity light exposure, terrestrial plants must develop stronger and more complex cell walls for self-protection than their aquatic plants (Boyce et al. 2004; Peter and Neale 2004). Cell walls are composed of various biological macromolecules, such as cellulose, lignin, and hemicellulose, which interlace to form the chief cell wall structural framework. Lignin and cellulose play key roles in plant life and growth processes. Previous study has revealed that stems with higher lignin content have greater mechanical strength and provide greater support to plants (Tripathi et al. 2003). Cellulose also plays a load-bearing role in the plant cell wall (Polko and Kieber 2019; Saxena and Brown 2005). Previous studies have shown that aquatic plants such as duckweed do not depend on vertical support structures, a characteristic which may be linked to genes involved in cell wall biosynthesis and lignification. Therefore, the structural differences between aquatic and terrestrial plants in the Araceae family may be related to their lignin and cellulose content (Wang et al. 2014). On the other hand, expansins are a class of cell wall loosening proteins that are involved in cell growth and root and root hair elongation. Therefore, investigating the functional differences in expansins between aquatic and terrestrial plants is particularly important (Cosgrove 2000).

Most True Araceae species develop starch-rich tubers that serve as vital energy reserves for their growth and development. The accumulation of starch is closely related to sugar transport proteins. The representative sugar transport proteins found in plants include Sugar transport protein (STP), Sucrose transporter (SUT), and Sugar will eventually be exported transporters (SWEET) transporter family proteins. These sugar transport proteins play essential roles in sugar-to-starch conversion, and the filling and yield of some plants depend on sugar transport (Wang et al. 2015). *HvSTP1* is mainly expressed in the barley endosperm region, where starch accumulates (Weschke et al. 2003). In rice, the expression level of the STP family gene *OsMST6* is greater in seeds during the initial grain filling and rapid growth stages (Wang et al. 2008). Several studies have shown that starch formation in wheat seed requires the involvement of SUT (Aoki et al. 2003; Deol et al. 2013). Moreover, inhibiting the expression level of the *OsSUT1* in rice reduces grain filling (Ishimaru et al. 2001; Aoki et al. 2002). In rice, knockout of *OsSWEET4* and *OsSWEET11* leads to weakened seed traits, suggesting that *OsSWEET4* and *OsSWEET11* have a significant impact on caryopsis development (Li et al. 2022). In maize, *ZmSWEET4c* maintains the development of large endosperm-storing starch in grain seeds (Sosso et al. 2015). In addition to its starch-rich tubers, the colored calla lily is known for its vibrant spathe, yet the mechanism behind spathe development remains unclear. MIKC^C^ genes play essential roles in plant flowering and floral organ identity (Barrero-Gil et al. 2021; Li et al. 2016; Theissen and Melzer 2007). Therefore, analyzing the MIKC^C^ genes in colored calla lily is crucial for gaining a deeper understanding of the mechanisms underlying spathe development.

In this study, we constructed a haplotype-resolved genome of the colored calla lily, highlighting substantial heterozygosity between homologous chromosomes. Comparative genomic analysis showed that transposon insertions are responsible for genome size variations among Araceae species. These species have undergone two closely-spaced WGD events before Araceae species divergence. Genes related to lignin synthase, cellulose synthase, expansin, and sugar transport proteins expanded in terrestrial True Araceae species, likely facilitating environmental adaptation and tuber starch synthesis. Additionally, we identified a crucial MIKC^C^ gene essential for spathe development. This study not only sheds light on the evolutionary history of Araceae species but also offers key genomic resources to support research and breeding for the colored calla lily.

## Results

### Genome assembly and quality assessment

**Fig. 1:**
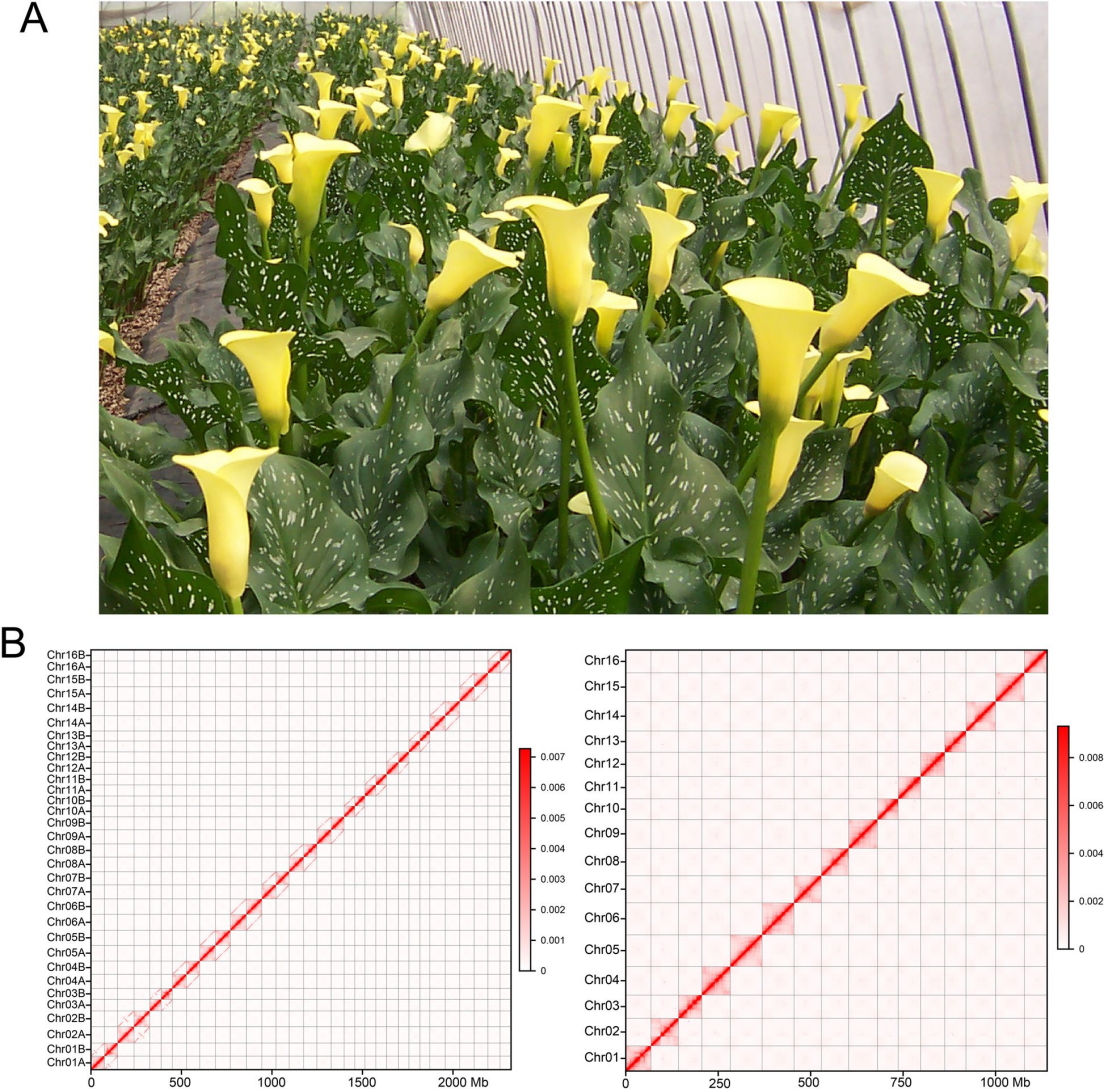
Genome assembly of colored calla lily. **A** Photograph of *Z. elliottiana* cv. ‘Jingcai Yangguang’. **B** Hi-C heatmap of the *Z. elliottiana* genome. The left figure shows the haplotype-resolved genome, and the right figure shows the chimeric genome

*Zantedeschia elliottiana* cv. ‘Jingcai Yangguang’ is a variety of colored calla lily with vibrant yellow spathes and leaves with numerous spots (Fig. 1A). Previous study using *k*-mer analysis determined that the haploid genome size is approximately 1.2 Gb (Wang et al. 2023). Through flow cytometry analysis, we estimated the haploid genome size of the colored calla lily to be approximately 1.22 Gb (Fig. S1; Table S1).

The genome was assembled using 29.83 × Oxford Nanopore Technologies (ONT) ultra-long reads (36.39 Gb), 36.92 × HiFi reads (43.93 Gb), 84.30 × Illumina paired-end reads (100.31 Gb) and 141.45 × Hi-C reads (168.18 Gb). Through genome assembly, we obtained contigs for both the haplotype-resolved genome (2,330 Mb) and the chimeric genome (1,202 Mb), with contig N50 values of 55.13 Mb and 73.20 Mb, respectively. Hi-C reads were used to anchor the contigs to the chromosomes, yielding two preliminary genome assemblies. For the haplotype-resolved genome, the 32 chromosomes were divided into two haplotypes, designated as haplotype A (Hap A) and haplotype B (Hap B), using chromosome-specific* k*-mers (Fig. S2; Table S2). This assembly resulted in a total genome size of 2.31 Gb. For the chimeric genome, the preliminary assembly contained only two gaps. By utilizing ONT ultra-long and PacBio HiFi reads, we successfully filled these gaps, resulting in a gap-free genome assembly with a total size of 1.15 Gb. The chimeric assembly produced 16 complete chromosomes, successfully identifying centromeric regions and detecting 30 telomeres (Fig. S3). Both assembled genomes display strong Hi-C interaction signals between chromosomes (Fig. 1B).

To assess the quality of the assembled genomes, we mapped RNA-seq reads from eight *Z. elliottiana* tissues (tuber, leaf, pistil, root, spathe, stamen, stem, and style) to the genomes. Approximately 95.08–97.26% of the reads were aligned to the haplotype-resolved genome, while around 92.49–95.30% were aligned to the chimeric genome (Table S3). Both assembled genomes demonstrated high mapping accuracy, with over 99% of the Illumina sequencing reads successfully aligned, covering more than 99% of the chromosomal sequences. The Benchmarking Universal Single-Copy Orthologs (BUSCO) evaluation revealed that both the haplotype-resolved genome and the chimeric genome have a high level of completeness for single-copy genes. The LTR assembly indices (LAIs) for the two genome assemblies were 18.84 and 19, respectively, demonstrating that the genome has high continuity. In summary, we have generated high-quality genome assemblies of *Z. elliottiana* (Table S4).

### Genome annotation

The proportion of repetitive sequences in the haplotype-resolved genome assembly and the chimeric genome assembly was 74.15% and 74.12%, respectively (Table S5). Repetitive sequences constituted a major portion of both assemblies, with 74.15% in the haplotype-resolved genome and 74.12% in the chimeric genome. LTR-RTs were the most prevalent type, accounting for 48.57% of the haplotype-resolved assembly and 47.13% of the chimeric assembly. Notably, Gypsy-type LTR-RTs were significantly more abundant than Copia-type LTR-RTs, accounting for 29.23% of the haplotype-resolved genome assembly and 28.39% of the chimeric genome assembly. A total of 62,609 protein-coding genes were predicted from the haplotype-resolved genome assembly, while 34,862 protein-coding genes were identified from the chimeric genome assembly (Table S6). To further illustrate the genomic features of the two assemblies, circos plots were generated displaying gene density, noncoding RNA density, Copia- and Gypsy-type LTR-RTs densities, DNA transposon density, gene expression levels and syntenic blocks (Fig. S4).

### Genome-wide comparison between HapA and HapB

**Fig. 2:**
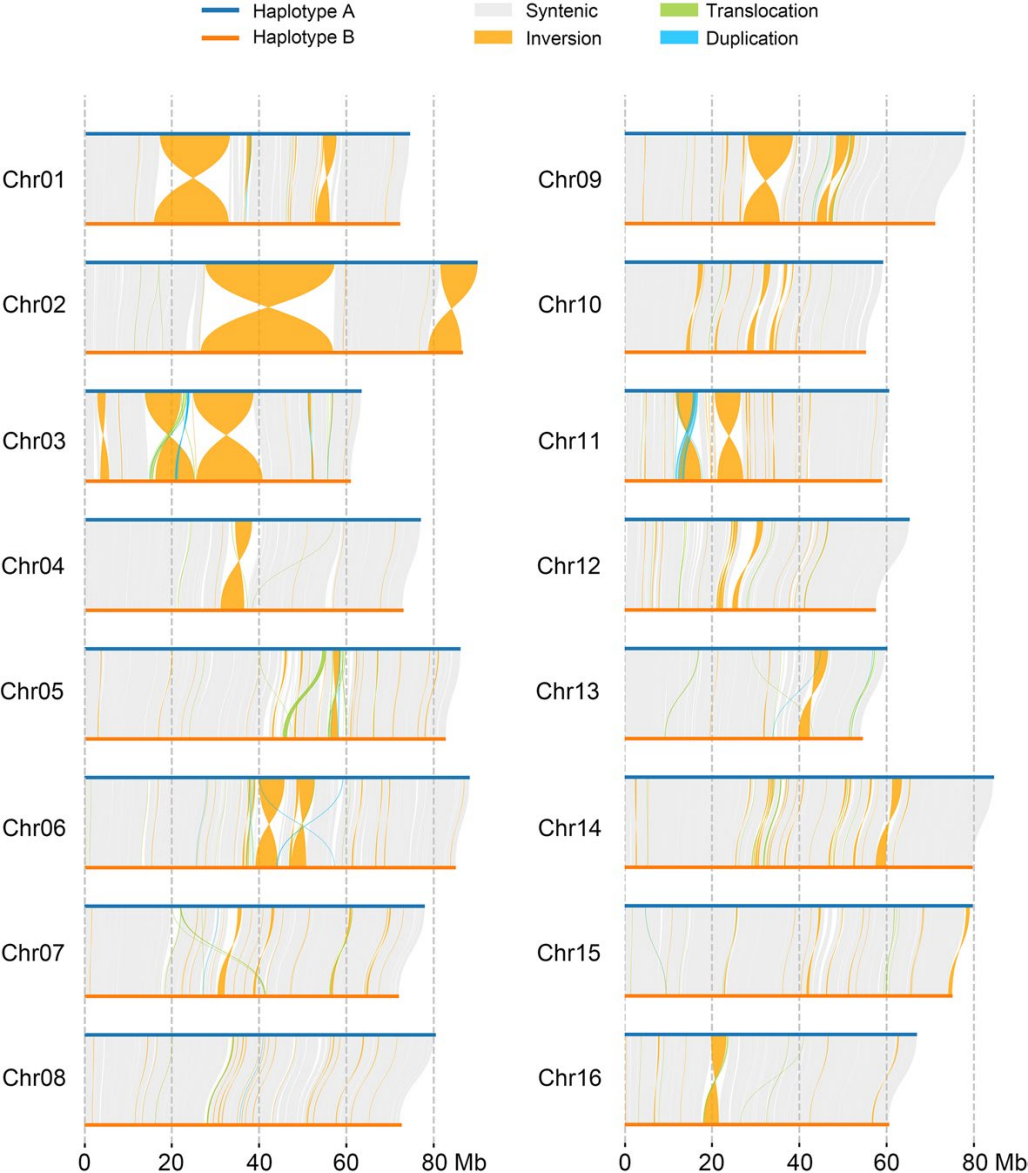
Genomic comparison between HapA and HapB. The blue and orange lines indicate HapA and HapB. Syntenic regions across the genomes are depicted by gray blocks. Inversions, translocations, and duplications are highlighted by orange, green, and blue curves, respectively

A comparative analysis between HapA and HapB revealed extensive genomic variations between the two haplotypes, identifying 1,227 syntenic regions that encompass approximately 1.82 Gb, highlighting a high degree of synteny between them (Fig. 2). We identified 246 inversions, with 155.09 Mb in haplotype A and 154.23 Mb in haplotype B, indicating significant structural rearrangements. Additionally, the genomes exhibited 613 translocations and 280 duplications, with lengths of 9.10 Mb and 2.03 Mb in haplotype A, and 9.32 Mb and 1.92 Mb in haplotype B, respectively (Table S7). Local Hi-C heatmaps demonstrated interaction signals between homologous chromosomes in inverted regions, confirming the accuracy of the assembly (Fig. S5). Further analysis of the impact of these structural variations on the phenotype of *Z. elliottiana* was conducted. Kyoto Encyclopedia of Genes and Genomes (KEGG) clustering analysis of the affected genes showed involvement in cyanoamino acid metabolism, glycerolipid metabolism, and fatty acid degradation pathways (Fig. S6). Among these, the *alcohol dehydrogenase* gene plays a crucial role in resistance to waterlogging, and structural variation in this gene may directly affect the adaptability of *Z. elliottiana* to its environment. Furthermore, *acylglycerol lipase* (*AGL*) and *diacylglycerol O-acyltransferase 1* (*DGAT1*) genes regulate the hydrolysis and synthesis of triglycerides, respectively, thereby influencing the cell membrane stability of *Z. elliottiana*. These findings reveal extensive structural variations between the two haplotypes of *Z. elliottiana*, providing valuable insights into its complex phenotype and genomic architecture.

### LTR-retrotransposon analysis in the Araceae family

**Fig. 3:**
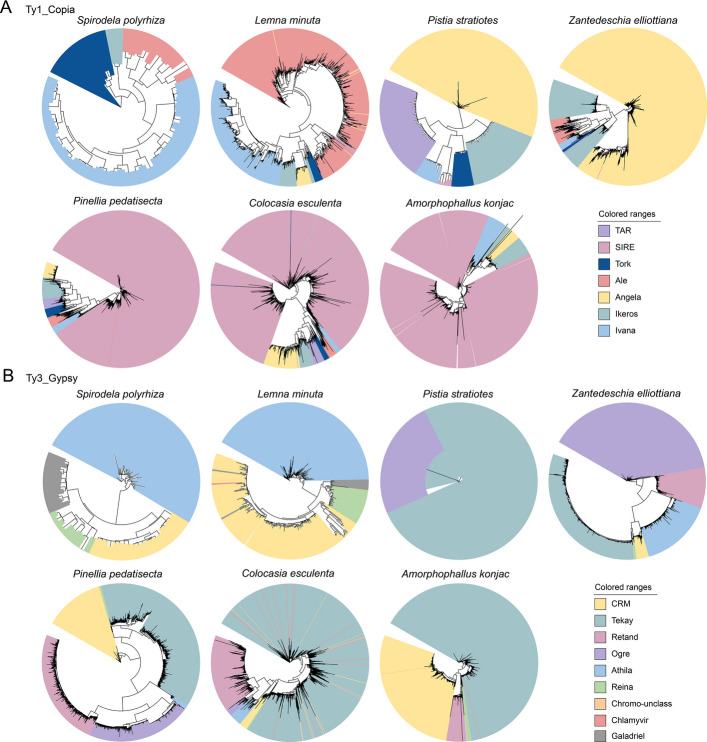
Transposon element analysis in Araceae species. **A** Phylogenetic tree of Ty1_Copia members in 7 Araceae species. **B** Phylogenetic tree of Ty3_Gypsy members in seven Araceae species

Previous study demonstrated that the extensive insertion of LTR-RTs leads to variation in genome size among Araceae species (Zhao et al. 2023). Within the LTR retrotransposon superfamily, Copia and Gypsy were positively correlated with genome size. We categorized all the identified LTR retrotransposons, providing detailed insights into the expansion of different subgroups. Specifically, Copia was divided into seven subgroups, and Ty3_Gypsy was also divided into nine subgroups. The expansion of Copia-type LTR-RTs in the Araceae species clearly varied among the different kinds of retrotransposons (Fig. 3A). In *P. stratiotes* and *Z. elliottiana*, the type of Copia that was amplified belonged to the Angela subgroup. In contrast, in the genomes of *A. konjac*, *C. esculenta*, and *P. pedatisecta*, the most predominant type was SIRE. Furthermore, the expansions of Gypsy-type LTR-RTs in Araceae species were relatively consistent, with the Tekay and Ogre subgroups exhibiting notable expansion trends (Fig. 3B). The types of LTR-RTs that expanded in species of the Lemnoideae subfamily, such as *S. polyrhiza* and *L. minuta*, exhibited significant differences. This may have contributed to the diversity of the species traits.

### Gene family expansion, contraction, and phylogenetic tree construction

**Fig. 4:**
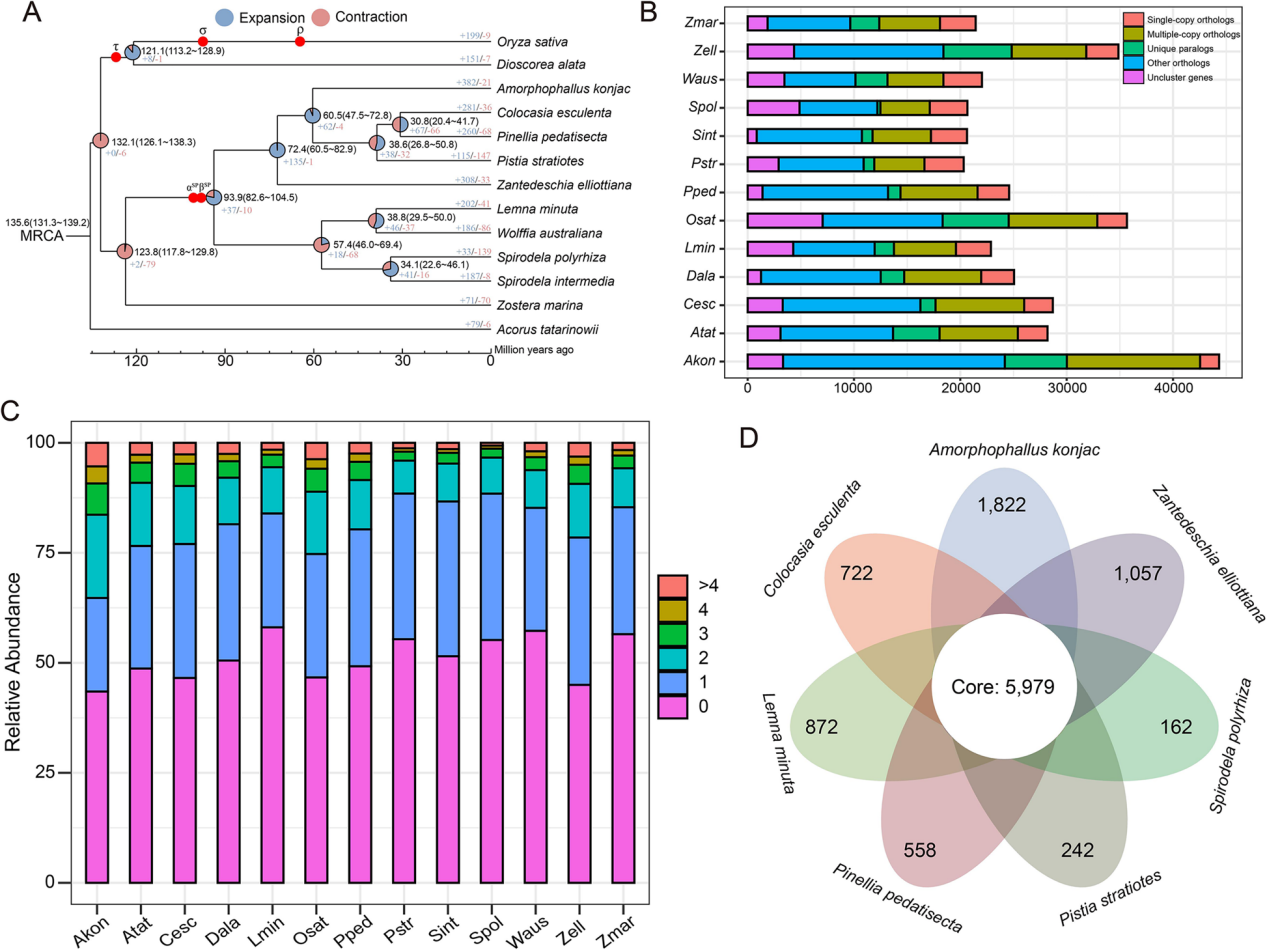
Comparative genomics analysis of Araceae species. **A** Phylogenetic tree, divergence times and gene family expansion and contraction among 13 species. Red circles indicate the occurrence of WGD events. **B** Distribution of various gene types, including unclustered genes, unique paralogs, other orthologs, single-copy orthologs, and multiple-copy orthologs, across the 13 plant species. **C** Distribution of gene family copy numbers among the 13 species. **D** Venn diagram showing the shared and specific gene families among *A. konjac*, *C. esculenta*, *P. pedatisecta*, *P. stratiotes*, *Z. elliottiana*, *S. polyrhiza* and* L. minuta*

Araceae is one of the most diverse monocotyledonous families and exhibits a rich array of morphological and ecological patterns. To investigate its phylogenetic relationships and evolution, we constructed phylogenetic trees of 13 species (including 9 Araceae species) using consensus single-copy genes. We used the oldest known species, *A. tatarinowii*, as an outgroup to study the differentiation time of monocotyledons. The ancestral species of Araceae appeared at 117.8–129.8 MYA and diverged into lemnoids and True Araceae at 82.6–104.5 MYA. The early differentiation of *Z. elliottiana* provides evidence that Araceae had the potential to survive on land as early as 60.5–82.9 MYA (Fig. 4A). *P. stratiotes*, a floating plant with the unique spathe of Araceae family, suggests that the differentiation of Araceae may have followed a process from floating on water to dwelling on land and then back to floating on water. In comparison to the other 13 species, Z. *elliottiana* exhibited expansion of 308 gene families and contraction of 33 gene families (Table S8). KEGG enrichment analysis of the expanded gene families in *Z. elliottiana* revealed the involvement of this gene in regulating oxidative phosphorylation (Table S9). On the other hand, the contracted gene families were primarily associated with sphingolipid metabolism (Fig. S7; Table S10).

The clustering analysis results of orthologs from 13 species showed that other orthologs constituted the largest proportion (Fig. 4B). A substantial number of unique paralogs have been identified in *Z. elliottiana*, directly revealing the significant differences between *Z. elliottiana* and other species in the Araceae family. Moreover, orthologs with zero copies were predominant, followed by orthologs with only one copy (Fig. 4C). To enhance our understanding of the specific and conserved gene families across Araceae species, we constructed a cluster analysis of gene families for seven Araceae species (*A. konjac*, *C. esculenta*, *P. pedatisecta*, *P. stratiotes*, *Z. elliottiana*, *S. polyrhiza*, and *L. minuta*) (Table S11). The results revealed 5,979 gene families conserved among these seven species within the Araceae family. and 1,057 gene families exclusive to *Z. elliottiana* (Figs. 4D, S8; Table S12). KEGG enrichment analysis revealed that conserved gene families in Araceae species are involved in nucleocytoplasmic transport and biosynthesis of amino acids (Table S13). Furthermore, we performed KEGG enrichment analysis of the specific gene families identified in *Z. elliottiana*, which indicated their participation in the flavone and flavonol biosynthesis and other types of O-glycan biosynthesis (Fig. S9; Table S14). Among these, the specific flavonol-3-O-glucoside L-rhamnosyltransferase catalyzes the rhamnosylation of flavonol glycosides, leading to the formation of compounds that exhibit antioxidant properties and enhance the defense response against biotic stress. Furthermore, the specific hydroxyproline O-Arabinosyltransferase plays a key role in catalyzing the arabinosylation of hydroxyproline residues. It is exclusively involved in the post-translational modification of extensins and may significantly contribute to the mechanical strength of the cell wall in colored calla lily.

### Whole-genome duplication and synteny analysis

**Fig. 5:**
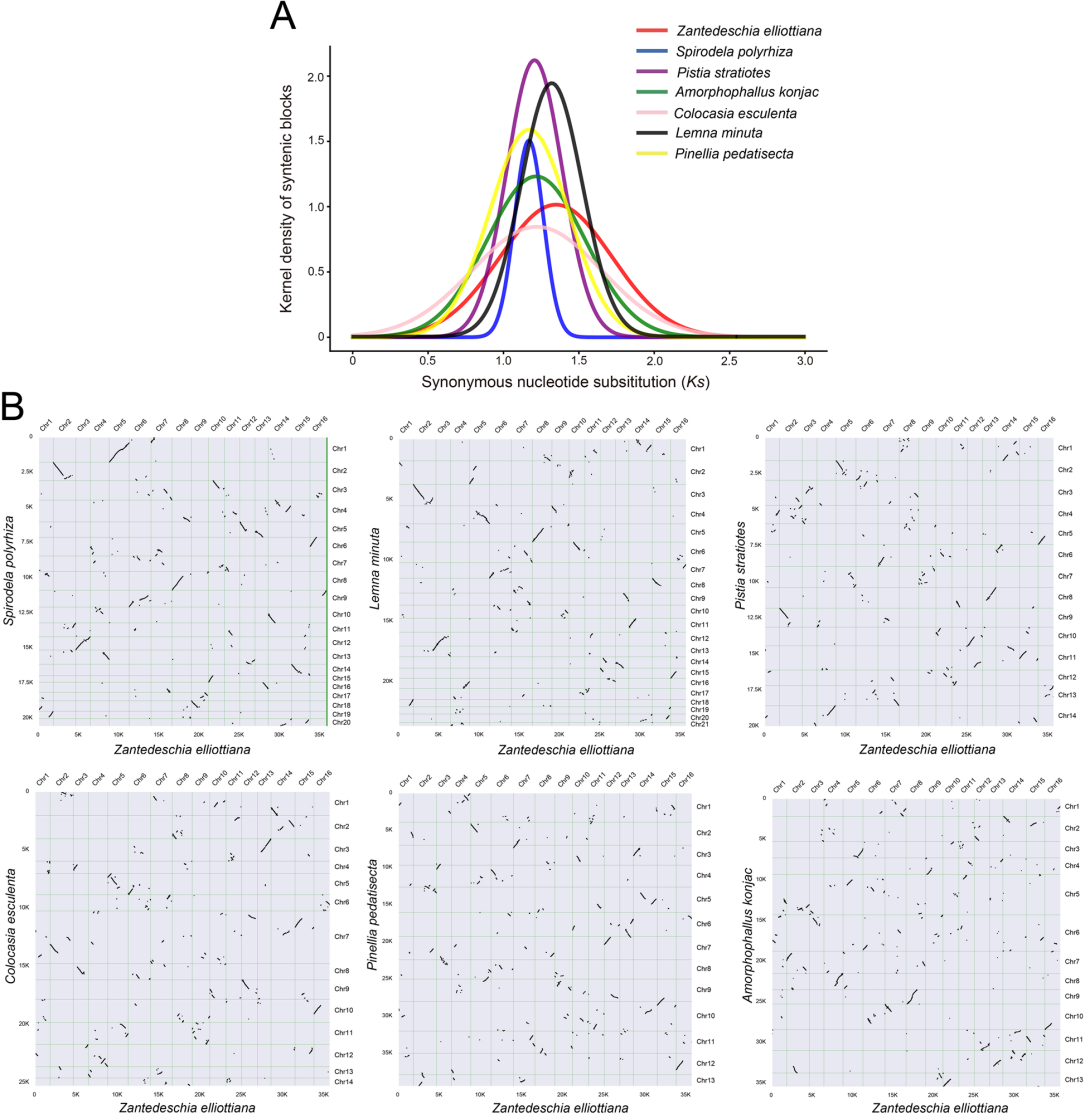
Whole-genome duplication analysis of Araceae species. **A** *Ks* distributions of *A. konjac*, *C. esculenta*, *P. pedatisecta*, *P. stratiotes*, *Z. elliottiana*, *S. polyrhiza* and *L. minuta*. **B** Identification of syntenic regions between Araceae species. Synteny analysis of *A. konjac*, *C. esculenta*, *P. pedatisecta*, *P. stratiotes*, *S. polyrhiza*, and *L. minuta* compared to *Z. elliottiana*

Previous studies have shown that the Araceae family underwent two WGD events, named α^SP^ and β^SP^, both occurring before its diversification and nearly simultaneously (Qian et al. 2022a; Wang et al. 2014; Clark and Donoghue 2018). The *Ks* distribution analysis revealed a single distinct peak for each species, with the peaks of most species tightly clustered. However, *L. minuta* and *Z. elliottiana* exhibited a slight shift, likely due to interspecific variation in base substitution rates (Fig. 5A). We hypothesized that the single peak in the *Ks* distribution analysis from the closely spaced occurrence of the two WGD events. Furthermore, syntenic regions were identified by comparing *Z. elliottiana* with *A. konjac*, *C. esculenta*, *P. pedatisecta*, *P. stratiotes*, *S. polyrhiza*, and *L. minuta*. Chromosomal rearrangements are widespread in Araceae species, contributing to the diversity of the Araceae family (Fig. 5B).

### Differences in morphogenesis and plant architecture among Araceae species

**Fig. 6:**
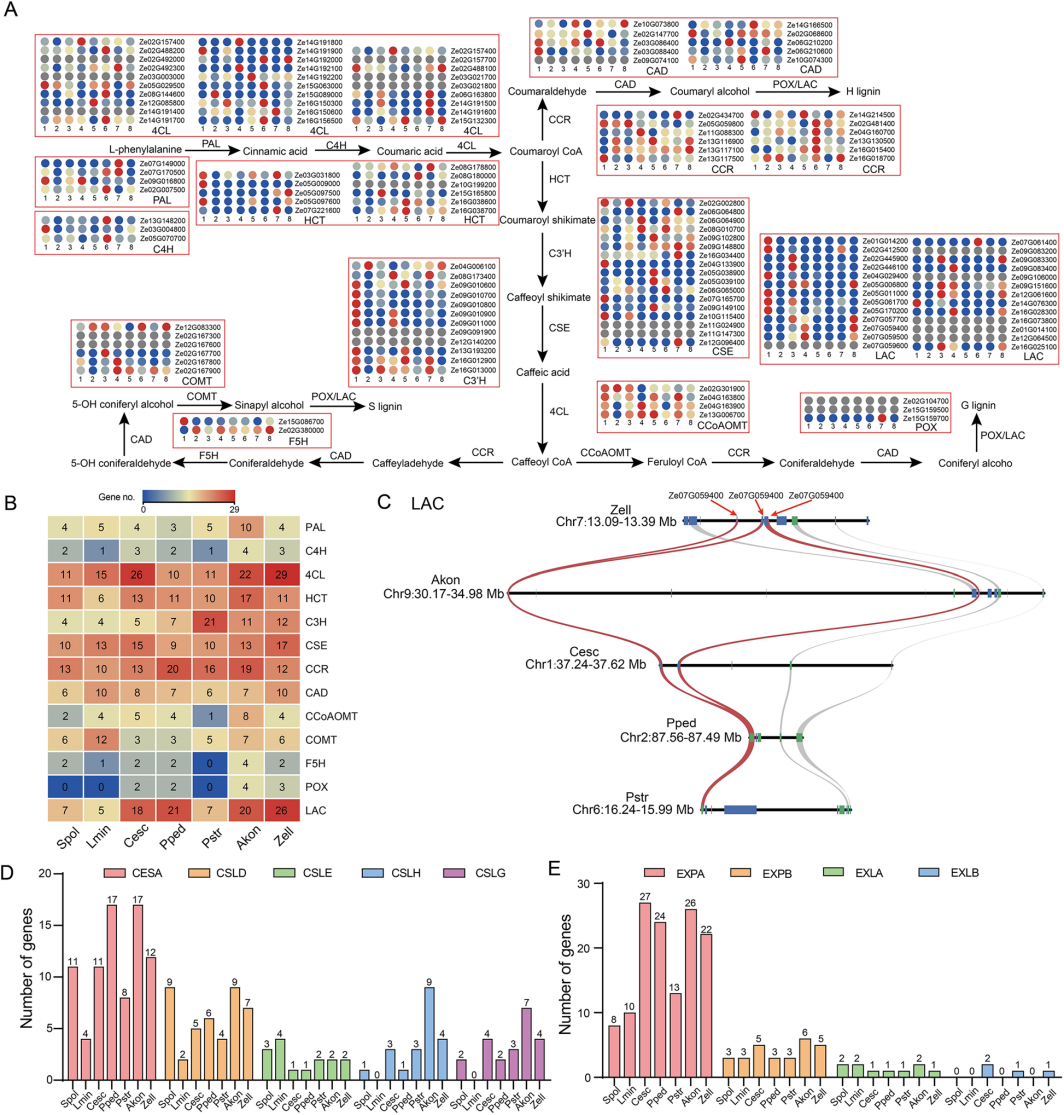
Differences in the numbers of lignin-, cellulose-, and expansin-related genes among the Araceae species. **A** Identification of genes involved in the lignin biosynthesis pathway in *Z. elliottiana*. 1–8 represent eight different tissues (1, tuber; 2, leaf; 3, pistil; 4, style; 5, root; 6, spathe; 7, stamen; 8, stem), and their expression levels are displayed in a heatmap. **B** The number of genes involved in lignin biosynthesis among Araceae species. **C** Collinearity analysis of LAC genes in True Araceae species. The green squares indicate genes on the forward strand, while the blue squares indicate genes on the reverse strand. Red lines indicate collinearity of LAC genes. **D** The number of cellulose synthase genes in Araceae species. (E) The number of expansin genes in Araceae species. Akon, *A. konjac*; Cesc, *C. esculenta*; Pped, *P. pedatisecta*; Pstr, *P. stratiotes*; Zell, *Z. elliottiana*; Spol, *S. polyrhiza*; Lmin,* L. minuta*

The evolutionary divergence of Araceae species resulted in differences in morphogenesis and plant architecture between lemnoids and True Araceae. Lignin, a common cell wall component in vascular plants, is also a key factor in plant growth and development (Fig. 6A). There are differences in the number of lignin biosynthetic genes between lemnoids and True Araceae. Peroxidase (POX) and laccase (LAC) promote the cross-linking of lignin in plant cell walls, increasing the stability and rigidity of the cell walls and enhancing the plant’s ability to respond to stress. The notable reduction in the number of POX/LAC genes in lemnoids in comparison to True Araceae provides compelling evidence that gene copy number plays a vital role as an evolutionary force influencing the development of traits (Fig. 6B). As an important enzyme in the process of lignin biosynthesis, caffeic acid-O-methyltransferase (CCoAOMT) plays a crucial role as a rate-limiting enzyme, and the gene copy number of *CCoAOMT* differs among different species in the Araceae family, as well as between lemnoids and True Araceae (Fig. 6B).

To examine the tissue-specific expression of lignin biosynthesis genes, a heatmap was generated to visualize the expression levels of these genes in eight different tissues of *Z. elliottiana* (Fig. 6A). Interestingly, we found that the laccase (LAC) genes are tandemly repeated on chromosome 7, and the highly expressed genes in the stem suggest their important role in plant structure. Through the identification of interspecies collinearity, it has been discovered that the tandem repeats of LAC genes are not conserved in True Araceae species. Localized gene repeats were present in *A. konjac*, *C. esculenta*, and *Z. elliottiana* (Fig. 6C).

True Araceae species are predominantly terrestrial, and their growth environment generally has less available nutrients and water. As a result, these plants require higher levels of cellulose in their cell walls to maintain their cell morphology, structure, and function. The number of cellulose synthase (CES) genes significantly increased in True Araceae species, yet these increases were not specific to any subfamily. An increase in the number of genes amplified the cellulose content in True Araceae species (Fig. 6D). The expansin (EXP) protein is crucial for plant cell growth and development, response to external stress, and improvement of plant adaptability. Our study revealed that the number of genes in the EXPA subfamily increased in the True Araceae species. In contrast, *P. stratiotes* did not exhibit a significant increase in the number of EXPA genes. This difference may be attributed to environmental factors (Fig. 6E).

### Starch accumulation in tubers

**Fig. 7:**
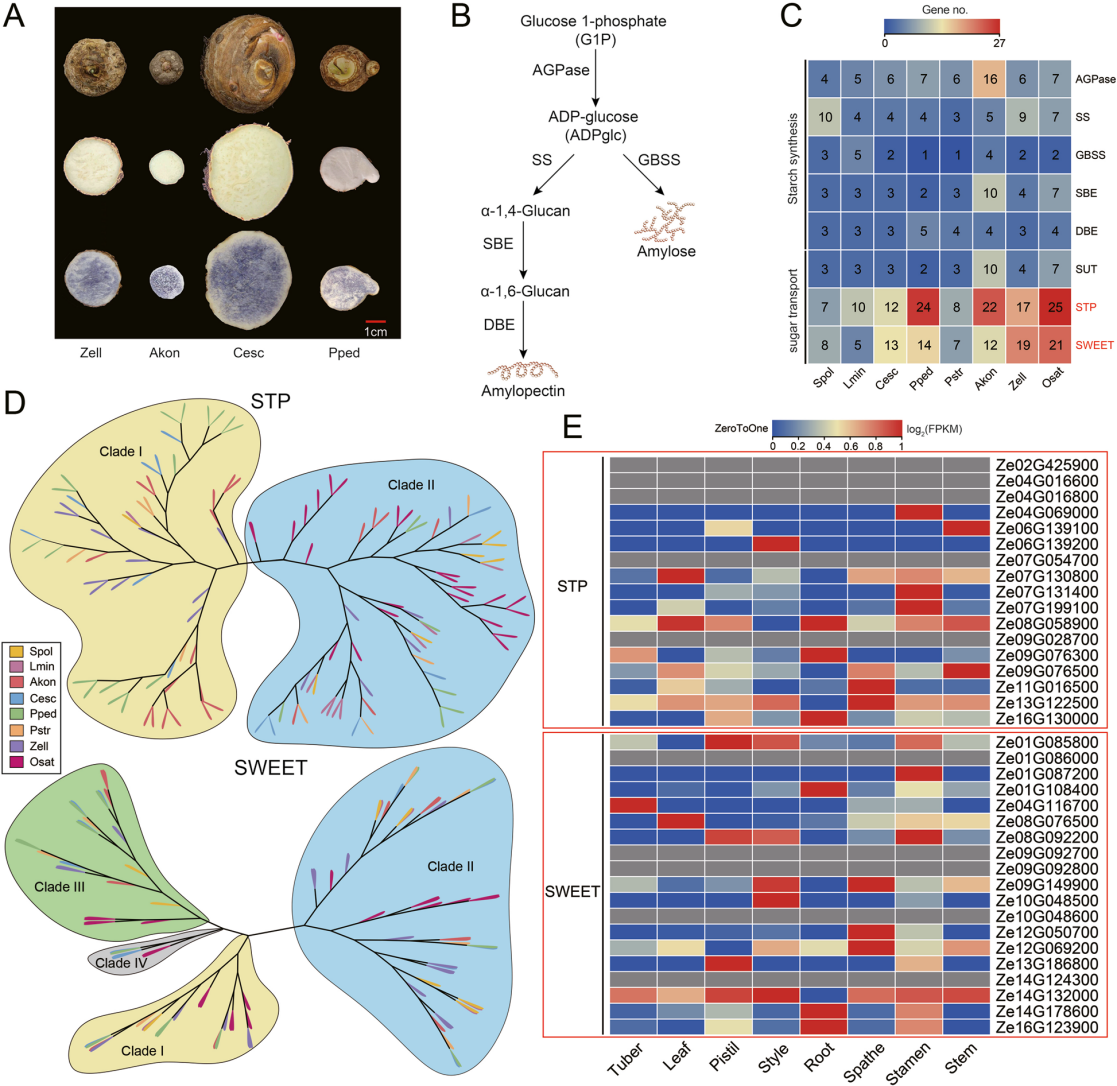
Starch accumulation in the tubers of True Araceae species. **A** Tuber phenotypes of *Z. elliottiana*, *A. konjac*, *C. esculenta*, and *P. pedatisecta*. Iodine staining of tuber cross-sections was performed to assess starch accumulation. **B** Schematic diagram of the starch biosynthesis pathway. **C** The number of genes related to starch biosynthesis and sugar transport in Araceae species. **D** Phylogenetic tree of the identified *STP* and *SWEET* genes. **E** Expression of the identified *STP* and *SWEET* genes in eight tissues of* Z. elliottiana*

*A. konjac* and *C. esculenta*, pivotal economic crops within the Araceae family, are predominantly cultivated for their starch-rich tubers. Due to their structural similarities with *A. konjac* and *C. esculenta*, *Z. elliottiana* and *P. pedatisecta* also demonstrate significant starch accumulation in their tubers (Fig. 7A). In contrast, this trait is notably absent in aquatic species such as *S. polyrhiza*, *L. minuta*, and *P. stratiotes* (Fig. S10). To determine the reasons underlying starch accumulation in tubers, we analyzed starch synthase and discovered no significant differences in starch synthase quantity among the eight species (Fig. 7B, C). Intriguingly, our results revealed a marked difference in the abundance of *STP* and *SWEET* genes (Fig. 7C). This disparity enables the prompt transport of sucrose synthesized in leaves to tubers, promoting starch accumulation. We constructed phylogenetic trees for the numerous identified *STP* and *SWEET* genes. Araceae species possess specific *STP* genes that have significantly expanded in species such as *A. konjac*, *C. esculenta*, *Z. elliottiana* and *P. pedatisecta* (Clade I). Concerning the *SWEET* genes, no difference in gene distribution existed among the clades, except for Clade IV (Fig. 7D). Nevertheless, among tuber-bearing True Araceae species, the *SWEET* gene family has experienced significant expansion. We determined the expression levels of the *STP* and *SWEET* genes in colored calla lily across diverse tissues through transcriptome analysis. Our results indicated that specific *STP* and *SWEET* genes were expressed across all tissues, suggesting they may play a key role in facilitating sugar transport throughout the plant (Fig. 7E).

### Spathe development of colored calla lily

**Fig. 8:**
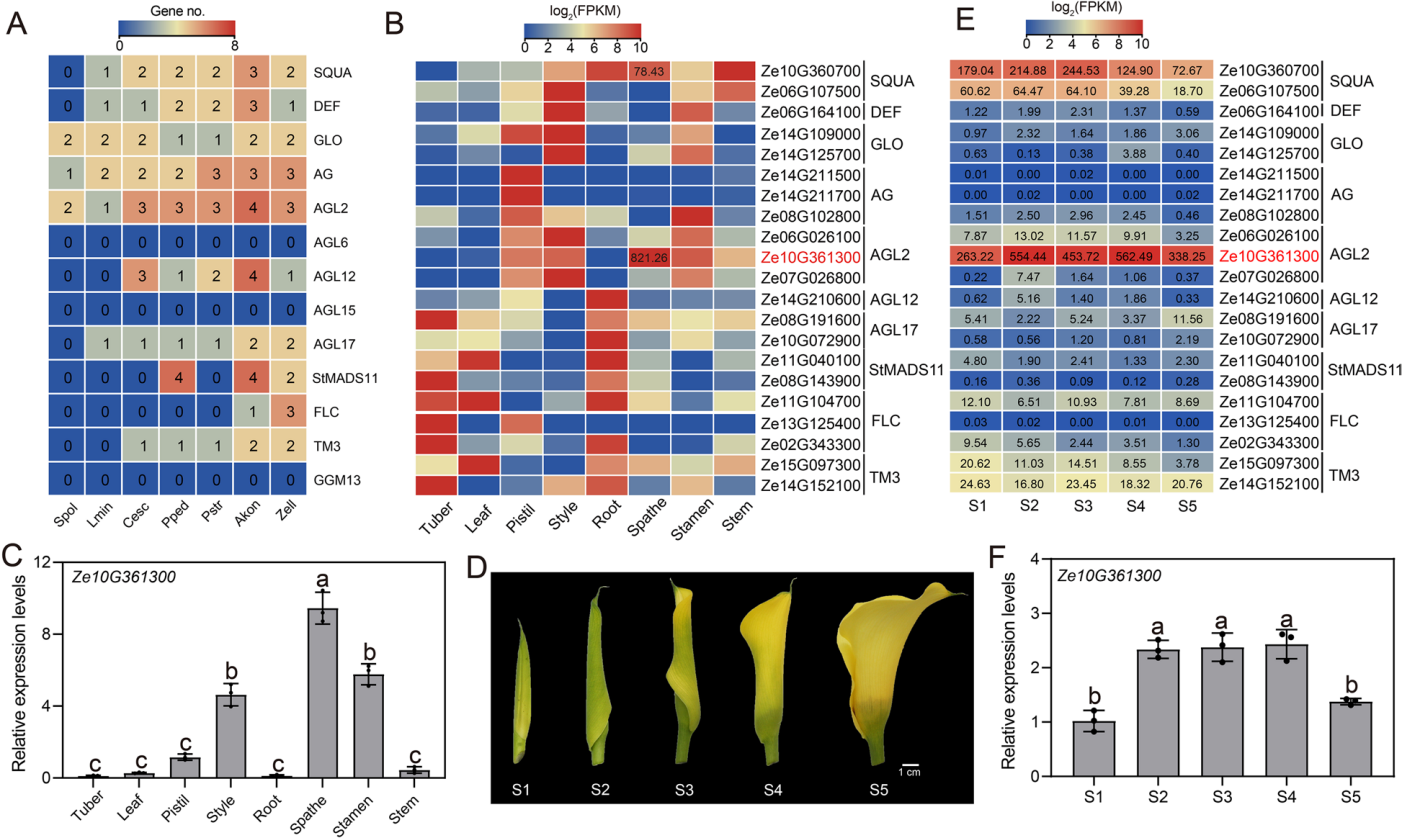
Spathe development in colored calla lily. **A** The number of MIKC^C^ genes in Araceae species. **B** Expression of identified MIKC^C^ genes in eight tissues of colored calla lily. Based on FPKM values, genes with high expression levels in the spathe were identified. Among them, *Ze10G361300* had the highest expression in the spathe and is denoted in red font. **C** The relative expression level of *Ze10G361300* in 8 tissues of colored calla lily. **D** Developmental stages of the colored calla lily spathe. **E** Expression levels of MIKC^C^ genes during the developmental stages of the colored calla lily spathe. **F** Expression levels of *Ze10G361300* in the spathe at different developmental stages were validated by RT-qPCR. Akon, *A. konjac*; Cesc, *C. esculenta*; Pped, *P. pedatisecta*; Pstr, *P. stratiotes*; Zell, *Z. elliottiana*; Spol, *S. polyrhiza*; Lmin,* L. minuta*

The spathe is a unique floral organ within the Araceae species and is characterized by a large and distinct bract surrounding the inflorescence. MIKC^C^ genes play crucial roles in the regulation of flowering time and establishment of floral morphology. Therefore, our objective was to identify and characterize MIKC^C^ genes to identify key genes involved in spathe development. We observed that lemnoid plants have considerably fewer MIKC^C^ genes than True Araceae species, which is consistent with their infrequent flowering characteristics. None of the Araceae species contained genes from the GGM13, AGL6, or AGL15 subfamilies, which mainly regulate seed dormancy, germination, and ovule development. In Arabidopsis, FLOWERING LOCUS C (FLC) serves as a key flowering suppressor that plays a crucial role in modulating flowering time. This effect is achieved by repressing the expression of *FLOWERING LOCUS T* (*FT*). Low temperatures can repress *FLC* expression, promoting Arabidopsis flowering. Interestingly, *FLC* genes were identified only in *Z. elliottiana* and *A. konjac*. We hypothesize that the Araceae family lost *FLC* genes during differentiation, which may explain their lack of dependence on low temperatures for the regulation of flowering (Fig. 8A).

We determined the expression levels of the MIKC^C^ genes in different colored calla lily tissues. Among these, the *AGAMOUS* (*AG*) genes (*Ze14G211500* and *Ze14G211700*) exhibited distinct tissue-specific expression, being exclusively expressed in the pistil. Simultaneously, we identified two AGL2 subfamily genes, *Ze06G026100* and *Ze07G026800*, which exhibited high expression levels in the style, while *Ze10G361300* displayed high expression levels in the spathe (Fig. 8B). We examined the expression level of *Ze10G361300* in 8 tissues to determine its role in spathe development. The results indicated that *Ze10G361300* was highly expressed in the spathe, consistent with the transcriptome data (Fig. 8C). To further validate whether *Ze10G361300* is involved in spathe development, five different developmental stages of colorful calla lily spathes were selected for transcriptome sequencing to determine the expression levels of MIKC^C^ genes (Fig. 8D). The results showed that *Ze10G361300* exhibited an increasing trend in expression during spathe development (Fig. 8E). The expression levels of *Ze10G361300* were further confirmed by RT-qPCR, consistent with the transcriptome data (Fig. 8F), further supporting its role as a key gene in spathe development.

## Discussion

The dynamic activity of TEs contributes significantly to the extensive diversity of plant genome sizes and structures (Bennetzen and Wang 2014). Notably, the variation in genome size among Araceae species is conspicuous, with the insertion of LTRs leading to considerable differences in genome size. In colored calla lily, Gypsy-type LTR-RTs, especially Tekay and Ogre, are predominant and have a direct impact on genome size (Fig. 3B). The expansion of Tekay-type LTR-RTs is highly conserved across species within the True Araceae species. Lemnoids tend to have smaller genomes and fewer genes, which may be due to the inactivity of LTR-RT insertions (Fig. 3). Interestingly, *P. stratiotes* also exhibits fewer LTR-RT insertions, which we speculate is due to its distinct survival environment. Species differentiation within the Araceae family appears to be relatively autonomous. As early as 132 MYA, monocotyledonous plants were separated from the Araceae lineage. The divergence of lemnoids occurred approximately 93.9 MYA, distinct from the differentiation of the True Araceae family. Intriguingly, despite being an aquatic plant, *P. stratiotes* is a True Araceae species, which directly demonstrates that early divergence does not dictate the ultimate habitat of a species. Compared to other True Araceae species, *Z. elliottiana* exhibited an earlier divergence time, occurring approximately 72.4 MYA. Previous studies have demonstrated that Araceae species underwent two WGD events, namely α^SP^ and β^SP^ (Qian et al. 2022a; Wang et al. 2014; Clark and Donoghue 2018). Our *Ks* distribution analysis revealed a single distinct peak for each species, which suggests that two WGD events may have occurred so closely together that they are difficult to distinguish (Fig. 5A).

Lignin and cellulose are key components that maintain the shape of plant cells (Polko and Kieber 2019; Saxena and Brown 2005). Our analysis revealed notable variations in the gene number associated with the lignin synthesis pathway among Araceae species, directly demonstrating that lignin synthesis is limited in aquatic plants. The *LAC* genes have undergone significant expansion across True Araceae species, influencing the accumulation of lignin. *POX* genes are completely absent in aquatic plants, which limits the diversity of lignin synthesis. Similar differences in the number of genes were also observed for cellulose synthase genes. These variations in the number of cellulose synthase genes directly impact the cellulose content within plants, consequently influencing cell morphology. Expansin is a type of plant cell wall loosening enzyme that can catalyze the depolymerization and repolymerization of cell wall polysaccharides, thereby participating in biological processes such as plant cell elongation, growth; cell division; and cell shape regulation (Cosgrove 2000). Upright growth is essential for the normal growth of terrestrial plants, and differences in the number of expansin genes directly contribute to this regulation. Expansin has a significant impact on regulating branching, participating in adverse responses, and controlling root morphology, thereby exerting a significant impact on root growth. We speculate that expansins also participate in the root development of terrestrial plants, thereby maintaining their ability to efficiently absorb nutrients from the soil. *A. konjac*, *C. esculenta* and *Z. elliottiana* all possess tubers in which starch accumulates, providing resources for plant growth and development. Sugar transport proteins play a crucial role in this process, with both the STP and SWEET gene families exhibiting expansion, which might be the primary reason for the substantial starch accumulation in the tubers. Building on this, we analyzed the WGD-mediated expansion of genes related to lignin synthase, cellulose synthase, expansin, and sugar transport proteins in the True Araceae family. The results revealed significant expansion of *LAC* and *CSEA* genes in terrestrial True Araceae species. Additionally, *EXPA* and *SWEET* genes showed expansion in True Araceae species, including *P. stratiotes* (Table S15). However, no relatively conserved expansion was detected for other genes, which suggests that although the WGD events are shared among Araceae species, they do not result in consistent expansion across all genes.

Additionally, we further analyzed the regulatory genes involved in the development of the colored calla lily spathe, where the AGL2 subfamily gene *Ze10G361300* may play a significant role. Meanwhile, we observed that the early spathes were similar to leaves in morphology, suggesting that they may originate from the same plant organ. The mechanism of floral organ identity remains highly complex, and future in-depth studies will further elucidate this mechanism in the Araceae family.

Our findings provide data for deciphering the evolution of the Araceae family, while the comparative genomics findings reveal the potential underlying molecular mechanisms of the differences in traits between aquatic and terrestrial plants within the Araceae family. This study provides theoretical genomic data for the diversification of Araceae species, laying the groundwork for subsequent comparative genomic analysis.

## Materials and methods

### Genome and transcriptome sequencing

For genome sequencing, leaves of the *Z. elliottiana* cv. ‘Jingcai Yangguang’ were selected (Fig. 1A) and cultivated in the greenhouse of the Yanqing Farm of the Beijing Academy of Agriculture and Forestry Sciences. The freash leaves were immediately placed in liquid nitrogen after collection and stored at −80 °C until use. Genomic DNA extraction was performed with the FastPure Plant DNA Isolation Mini Kit (Vazyme) following the manufacturer's guidelines. The concentration and purity of the isolated DNA were assessed using a NanoDrop 2000 (Thermo Scientific) and gel electrophoresis. High-quality DNA was used for genomic library construction. Library preparation and sequencing were conducted at Novogene Co., Ltd., in Beijing; PacBio HiFi, Hi-C, and Illumina sequencing; and at Grandomics Co., Ltd., in Wuhan, which was responsible for ONT ultra-long sequencing. For Hi-C sequencing, the same leaf sample described earlier was used to produce the Hi-C library. Briefly, nuclear DNA was crosslinked with formaldehyde and then digested using the restriction enzyme *Dpn*II. Biotinylated nucleotides were incorporated at the fragmented DNA ends. This was followed by an enrichment process and size selection, targeting fragments approximately 500 bp. The libraries were subsequently processed on the NovaSeq 6000 platform.

Transcriptome sequencing was performed on eight different tissues: tuber, leaf, pistil, root, spathe, stamen, stem, and style. RNA was extracted from these tissues via the Tiangen RNAprep Pure Plant Kit (TIANGEN). The tissues were pulverized with liquid nitrogen, and the lysis buffer was used for RNA extraction. Library construction and sequencing services were provided by Novogene Co., Ltd., in Beijing.

### Flow cytometry analysis

Flow cytometry was used to estimate the genome size of the *Z. elliottiana* cv. ‘Jingcai Yangguang’. For this purpose, leaves were immersed in 500 μL of CyStain PI Absolute P Nuclei Extraction Buffer (Tanaka et al. 2006) (Sysmex Partec) and finely chopped with a razor blade, followed by filtration through a 50 μm mesh. The resultant cell suspension was then mixed with 2,000 μL of CyStain PI Absolute P Staining Buffer (Sysmex Partec) with RNase and incubated in the dark for 30 min. Analysis was conducted using a CyFlow Space Flow Cytometer (Sysmex Partec). Tomato (*Solanum lycopersicum*) was used as the reference species for genome size, with a known genome size of 799.09 Mb. The nuclear suspension from the flow cytometry analysis was evaluated using FCSExpress software (v.3).

### Genome assembly and gap filling

The assembly of contigs derived from ONT ultra-long and PacBio HiFi reads was conducted using hifiasm (v.0.19.8) (https://github.com/chhylp123/hifiasm) (Cheng et al. 2021), with parameters -l 0 and 3 used to obtain haplotype-resolved and chimeric contigs, respectively. The assembly efficiency was gauged by aligning the Hi-C reads to the contigs using HICUP (v.0.7.3) (Wingett et al. 2015). Subsequently, YaHS (v.1.1) (Zhou et al. 2023) anchored these contigs into chromosomes. Manual corrections to the assembled genome were made using Juicebox (v.1.11.08) (Durand et al. 2016). The quarTeT (v.1.2.1) (Lin et al. 2023) was used to fill these gaps, utilizing ONT ultra-long and PacBio HiFi reads. The identification and clustering analysis of chromosome-specific *k*-mers were performed using SubPhaser (v.1.2.6) (Jia et al. 2022).

The BUSCO (v.5.4.5) (Simão et al. 2015) and LAI (LTR_retriever; v.2.9.0) (Ou et al. 2018) were applied to provide a comprehensive assessment of genome completeness and continuity.

### Identification of telomeres and centromeres

Initially, the telomeres of the draft assembly were pinpointed using 7-mer repeats (CCCTAAA/TTTAGGG). Furthermore, these 7-mer repeats were utilized in analyzing contigs from NextDenovo (v.2.5.2) (Hu et al. 2024), HiCanu (v.2.2) (Nurk et al. 2020), and hifiasm (v.0.19.8) (Cheng et al. 2021), leading to the discovery of previously undetected telomeres. The quarTeT (v.1.2.1) (Lin et al. 2023) was also used for telomere identification. Due to limitations in the data, two telomeres on Chr04 and Chr13 could not be filled. TRF (v.4.09.1) was utilized for the identification and classification of satellite, minisatellite, and microsatellite sequences within the chimeric genome (Benson 1999). TRF was performed under the default parameters ‘2 7 80 10 50 500 -f -d -m’, and the annotated results were consolidated using TRF2GFF (https://github.com/Adamtaranto/TRF2GFF). We excluded tandem repeats that had fewer than five copies or were redundant. The classification of sequences into microsatellites (less than 10 bp), minisatellites (10–100 bp), and satellites (over 100 bp) was based on their length. The approximate location of the centromeres was inferred from the EDTA (v.2.1.0) (Su et al. 2021) and TEsorter (v.1.4.6) (Zhang et al. 2022). The IGV (v.2.18.0) (Robinson et al. 2011) was used to identify overlaps between regions lacking transposable element annotations and those enriched with tandem repeats, enabling the identification of centromeric regions.

### Repeat identification and genome annotation

For repeat identification, a comprehensive annotation pipeline integrating both de novo and homology-based methods was utilized. The homology approach involved aligning sequences with the RepBase database (http://www.girinst.org/repbase), followed by prediction using RepeatProteinMask. De novo annotation was facilitated by constructing a library with tools such as LTR_FINDER (https://github.com/xzhub/LTR_Finder) (Xu and Wang 2007), RepeatScout (v.1.0.6) (Price et al. 2005), and RepeatModeler (v.2.0.6) (Flynn et al. 2020) (http://www.repeatmasker.org/RepeatModeler.html), followed by annotation using RepeatMasker (http://repeatmasker.org/) (v.4.0.6) (Nishimura 2000).

Gene structure annotation was performed via a multifaceted strategy. Proteins from Araceae species including *A. konjac*, *C. esculenta*, *L. minuta*, *S. polyrhiza*, *P. stratiotes*, and *P. pedatisecta* were mapped to their genomes using WUblast (v.2.0) (Bedell et al. 2000). GeneWise (v.2.4.1) (Birney et al. 2004) was used to predict the gene structures in the genomic regions identified by WUblast (v.2.0) (Bedell et al. 2000). The gene structures generated by GeneWise were referred to as the Homo-set. Additionally, gene models were produced by PASA (v2.5.3) (Haas et al. 2008), which served as training data for de novo gene prediction programs. Five de novo gene prediction programs, namely, AUGUSTUS (v.2.5.5) (Stanke et al. 2006), Genscan (v.1.0) (Burge and Karlin 1997), Geneid (v.1.4.5) (Guigo 1998), GlimmerHMM (v.3.0.1) (Majoros et al. 2004) and SNAP (Korf 2004), were used to predict coding regions within the repeat-masked genome. The transcript-based annotation involved aligning the clean data to the genome assembly using TopHat (v.2.0) (Kim et al. 2013) and Cufflinks (v.2.1.1) (Trapnell et al. 2012), with EVidenceModeler (v.2.1.0) (Haas et al. 2008), which integrates these results into a unified gene annotation set.

Functional annotation of the predicted proteins was conducted via searches across the NR, InterPro, KEGG, and UniProt (Swiss-Prot) (v.2023_01) databases, with Gene Ontology annotations derived using InterProScan (v5.0) (Mulder and Apweiler 2007).

### Genome collinearity and gene family clustering analysis

Genome-wide comparison analysis was conducted using SyRI (v.1.7.0) (Goel et al. 2019) to assess differences between HapA and HapB. Gene collinearity was analyzed using JCVI (Tang et al. 2024). The “python -m jcvi.compara.catalog ortholog –cscore 0.9” package was used to find the gene pairs. A total of 13 species, *Z. elliottiana*, *P. pedatisecta*, *W. australiana, S. polyrhiza*, *P. stratiotes*, *L. minuta*, *C. esculenta*, *A. tatarinowii*, *S. intermedia*, *D. alata*, *Z. marina*, *A. konjac* and *O. sativa* were analyzed with OrthoFinder (Emms and Kelly 2015) to construct the gene families.

### Construction of the phylogenetic tree and estimation of divergence times

A set of single-copy genes consisting of 237 genes from 13 species was chosen for analysis. Initially, the gene sequences (237 sequences per species) were aligned using MUSCLE (v.5.1) (Edgar 2004). Subsequently, a maximum-likelihood tree was constructed using RAxML (v.8.0.19) (Stamatakis 2014), and 100 bootstrap replicates were conducted to assess the accuracy of the phylogenetic tree. Divergence times were estimated using the MCMCTree program in PAML (v.4.10.7) (Yang 2007).

### Expansion and contraction of gene families

The likelihood model implemented in CAFE (v.4.2.1) (https://github.com/hahnlab/CAFE) was employed for our analysis. The branch lengths of the phylogenetic tree were considered to determine the significance of changes in gene family size.

### Whole-genome duplication analysis

The *Ks* analysis was performed using WGDI (Sun et al. 2022), where *Ks* values were extracted under the PAML yn00 NG model for the WGDI-KsPeaks analysis (Yang 1997). The WGDI-PeaksFit was applied in median mode, and figure was generated using WGDI-KsFigure.

#### Comprehensive analysis of lignin biosynthesis genes

The identification of all genes involved in the lignin synthesis pathway was accomplished using BlastKOALA (https://www.kegg.jp/blastkoala/) (Kanehisa et al. 2016). Protein sequences from each species were annotated and assigned to the KEGG orthology database. Proteins homologous to the identified proteins were searched against the Swiss-Prot database using BLASTP (v.2.15.0) (McGinnis and Madden 2004). The annotation was manually verified. Transcriptome analysis across various tissues involved aligning clean data to the genome with HISAT2 (v.2.2.1) (Kim et al. 2019) and quantifying gene expression using featureCounts (v.2.0.6) (Liao et al. 2013). The FPKM value was utilized to assess gene expression across different tissues, and the expression levels were visualized as heatmap using TBtools (Chen et al. 2020).

#### Identification of expansin-related proteins and cellulose synthase genes

Identification of expansin-related proteins and cellulose synthase genes in each species was conducted using HMMER (v.3.3.2) (Wheeler and Eddy 2013) and BLASTP (v.2.15.0) (McGinnis and Madden 2004). For the identification process, two functional domains (PF01357 and PF03552) from InterPro were specifically employed to pinpoint expansin-related proteins and cellulose synthase genes. We utilized protein sequences of expansin-related and cellulose synthase from Arabidopsis as reference genes to facilitate searches of the genes in each species. The reliability of the results was confirmed for the proteins identified by both methods via an NCBI Conserved Domain Search (https://www.ncbi.nlm.nih.gov/Structure/cdd/wrpsb.cgi).

#### Comprehensive analysis of genes associated with tuber starch biosynthesis

The identification method for starch biosynthesis genes was the same as that for lignin synthesis genes. The protein sequences of STP and SWEET from Arabidopsis were utilized to identify corresponding genes within each species*.* The identified genes were ultimately utilized to construct a phylogenetic tree to assess gene amplification across various species. The protein sequence alignments were performed using MUSCLE (v.5.1) (Edgar 2004), while the construction of phylogenetic trees was performed via the maximum-likelihood method through IQ-TREE (Minh et al. 2020).

#### Comprehensive analysis of the MIKC^C^-type MADS-box genes

The gene identification was based on previous study (Wang et al. 2022). The relative expression levels in different tissues were verified by qRT‒PCR. The *ACTIN* gene from *Z. elliottiana* was used as the internal reference gene (Table S16) (Wang et al. 2024).

## Supplementary Information

Supplementary Material 1. Figure S1. Evaluating the genome sizes of *Z. elliottiana* using flow cytometry. *Solanum lycopersicum was* used as a control to calculate the genome size of *Z. elliottiana.* Measurements were performed with three biological replicates.

Supplementary Material 2. Figure S2. Assigning chromosomes to haplotypes using specific *k*-mers. A Principal component analysis of chromosome-specific *k*-mers. B Clustering analysis of differential *k*-mers.

Supplementary Material 3. Figure S3. The locations of centromeres and telomeres on each chromosome in the chimeric genome.

Supplementary Material 4. Figure S4. Circos plot of *Z. elliottiana* genomes. A The left panel shows the haplotype-resolved genome. B The right panel shows the chimeric genome. (a) Chromosome length and number. (b) Orange and blue lines represent gene density and noncoding RNA density, respectively. (c) Density of Copia-type LTR-RTs. (d) Density of Gypsy-type LTR-RTs. (e) DNA transposon density. (f) Gene expression levels. (g) syntenic blocks.

Supplementary Material 5. Figure S5. Local Hi-C heatmaps were used to validate the accuracy of genomic variation detection between homologous chromosomes.

Supplementary Material 6. Figure S6. KEGG enrichment analysis of genes affected by genomic variation between the two haplotypes of *Z. elliottiana* genome.

Supplementary Material 7. Figure S7. KEGG enrichment analysis of expanded/contracted gene families in *Z. elliottiana.*

Supplementary Material 8. Figure S8. Gene family clustering analysis of 7 Araceae species. The species included *A. konjac*, *C. esculenta*, *P. pedatisecta*, *P. stratiotes*, *Z. elliottiana*, *S. polyrhiza*, and *L. minuta*.

Supplementary Material 9. Figure S9. KEGG enrichment analysis of conserved gene families in Araceae and specific gene families in *Z. elliottiana* from gene family cluster analysis.

Supplementary Material 10. Figure S10. Photographs of *P. stratiotes* used to reveal floating aquatic plants in the True Araceae family.

Supplementary Material 11. Table S1. Genome size estimation of *Z. elliottiana* by flow cytometry. Table S2. Chromosome lengths (bp) and number of protein-coding genes for each chromosome of the *Z. elliottiana*. Table S3. Mapping rate of transcripts from different tissues. Table S4. Summary of the *Z. elliottiana* genome. Table S5. Classification of repetitive sequences in Z. elliottiana genome. Table S6. General statistics of predicted protein-coding genes. Table S7. Summary of variation types between HapA and HapB. Table S8. Comparative genomic analysis of expansion/contraction genes in *Z. elliottiana*. Table S9. KEGG annotation of expansion genes in *Z. elliottiana*. Table S10. KEGG annotation of contraction genes in *Z. elliottiana*. Table S11. Fundamental information on gene family clustering among seven Araceae species. Table S12. Cluster analysis of gene families among seven Araceae species. Table S13. KEGG enrichment analysis revealed conserved gene families in Araceae. Table S14. KEGG enrichment analysis revealed the gene families specific to *Z. elliottiana* in the Araceae. Table S15. WGD-mediated expansion of genes associated with lignin synthase, cellulose synthase, expansin, and sugar transport proteins in the True Araceae. Table S16. The sequences of primers used in this study.

## Data Availability

The raw data utilized for genome assembly have been deposited in the NCBI under BioProject accession numbers PRJNA1060881 and PRJNA956997. The RNA-seq data can also be found at NCBI under BioProject accession numbers PRJNA957064 and PRJNA1226726. Additionally, the genome assembly and annotation files are available at 10.6084/m9.figshare.24941703.

## Abbreviations

AG: AGAMOUS
BUSCO: Benchmarking Universal Single-Copy Orthologs
CCoAOMT: Caffeic acid-O-methyltransferase
CES: Cellulose synthase
EXP: Expansin
FLC: FLOWERING LOCUS C
FPKM: Fragments Per Kilobase of transcript per Million mapped reads
FT: FLOWERING LOCUS T
KEGG: Kyoto Encyclopedia of Genes and Genomes
LAI: LTR assembly index
LAC: Laccase
LTR: Long-terminal repeat
LTR-RTs: Long-terminal repeat retrotransposons
MIKC^C^: MIKC^C^-type MADS-box
MYA: Million years ago
ONT: Oxford Nanopore Technologies
POX: Peroxidase
STP: Sugar transport protein
SUT: Sucrose transporter
SWEET: Sugar will eventually be exported transporters
WGD: Whole-genome duplication
