# Supplementary figures and images for: Haplotype-resolved genome assembly of the colored calla lily (Zantedeschia elliottiana) provides new insights into the evolution of the family Araceae
Source: Mol Hortic. 2026 Feb 9;6:12. doi: 10.1186/s43897-025-00192-w (PMC12884619; doi:10.1186/s43897-025-00192-w)

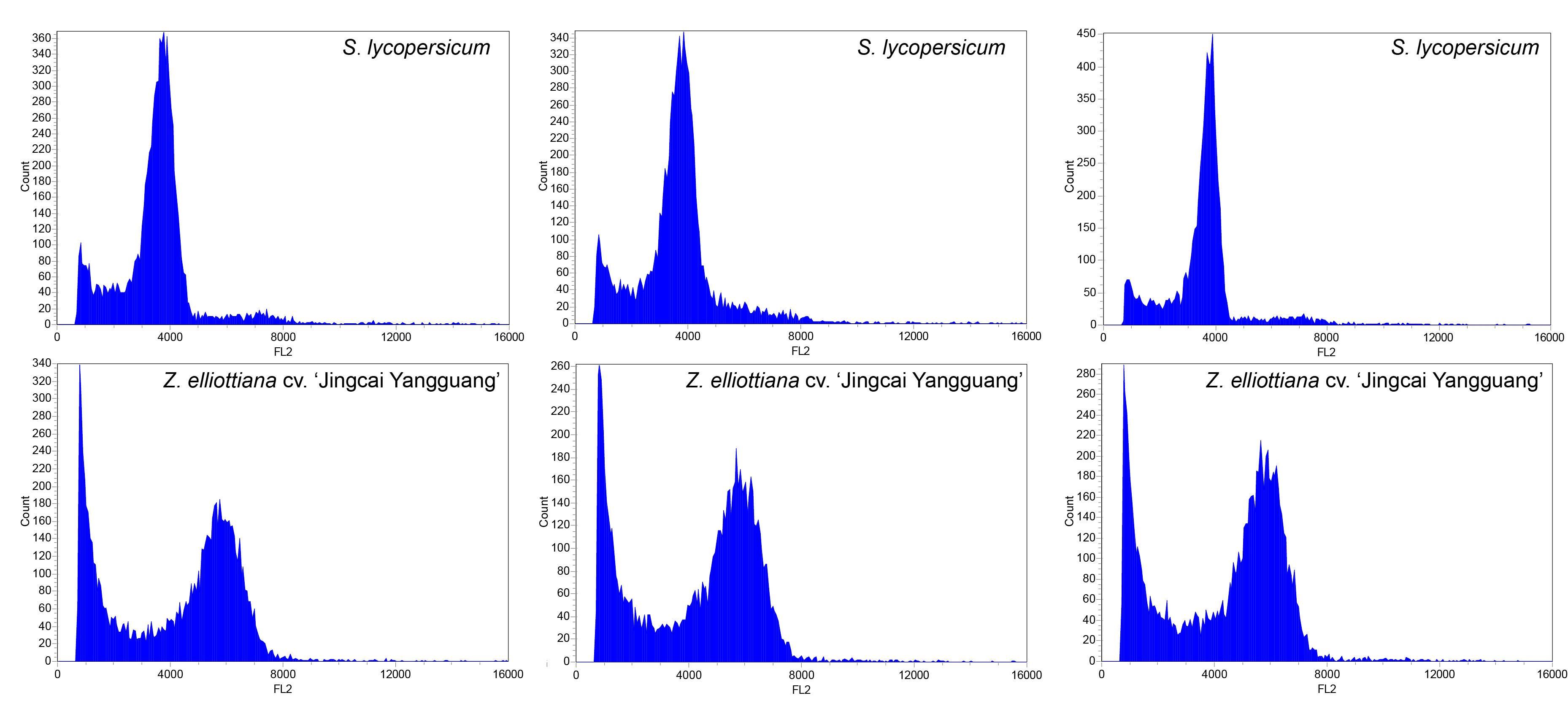

Supplement: Supplementary file 1 — Supplementary Material 1. Figure S1. Evaluating the genome sizes of Z. elliottiana using flow cytometry. Solanum lycopersicum was used as a control to calculate the genome size of Z. elliottiana. Measurements were performed with three biological replicates. [file 43897_2025_192_MOESM1_ESM.jpg]

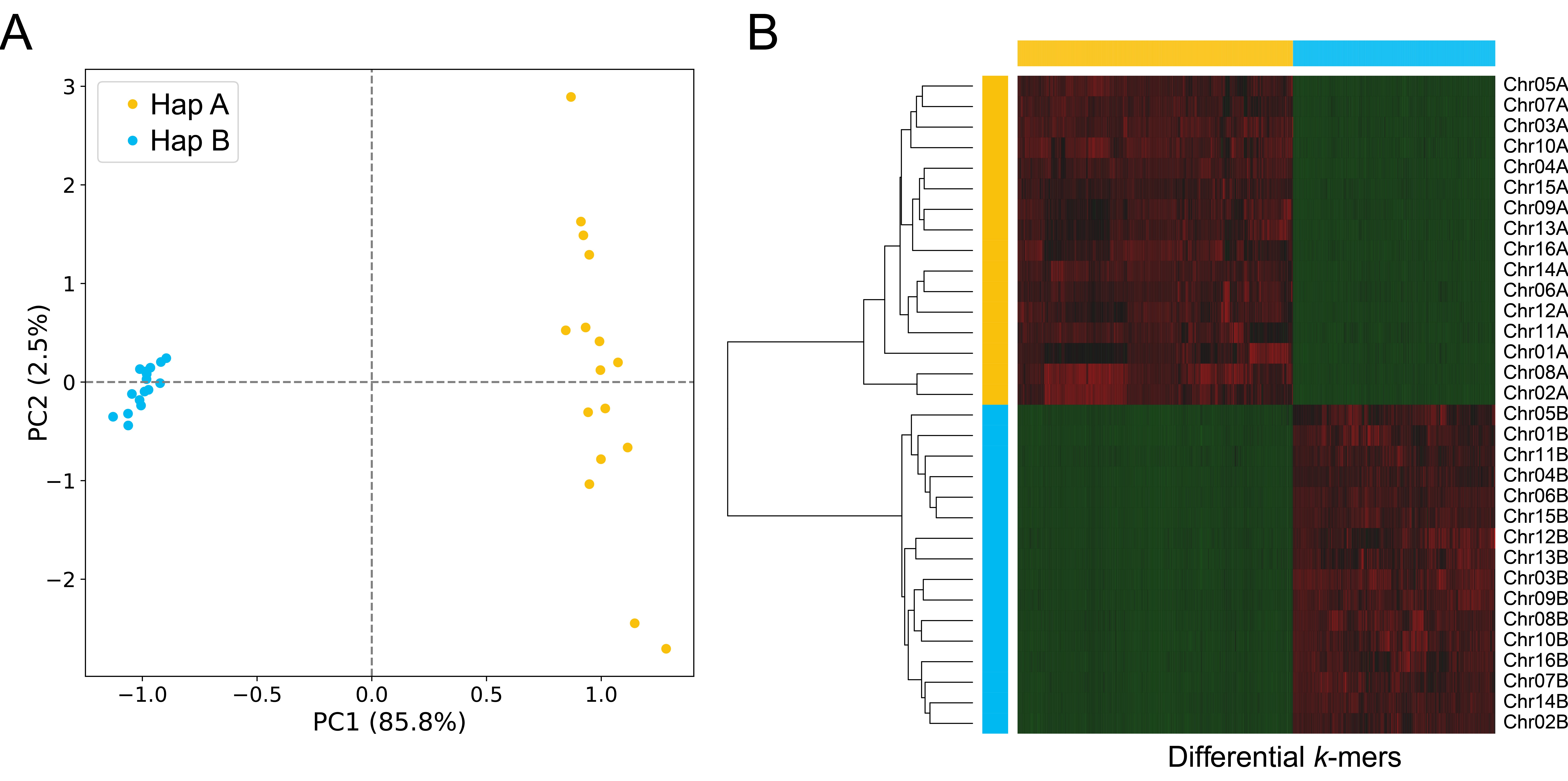

Supplement: Supplementary file 2 — Supplementary Material 2. Figure S2. Assigning chromosomes to haplotypes using specific k-mers. A Principal component analysis of chromosome-specific k-mers. B Clustering analysis of differential k-mers. [file 43897_2025_192_MOESM2_ESM.jpg]

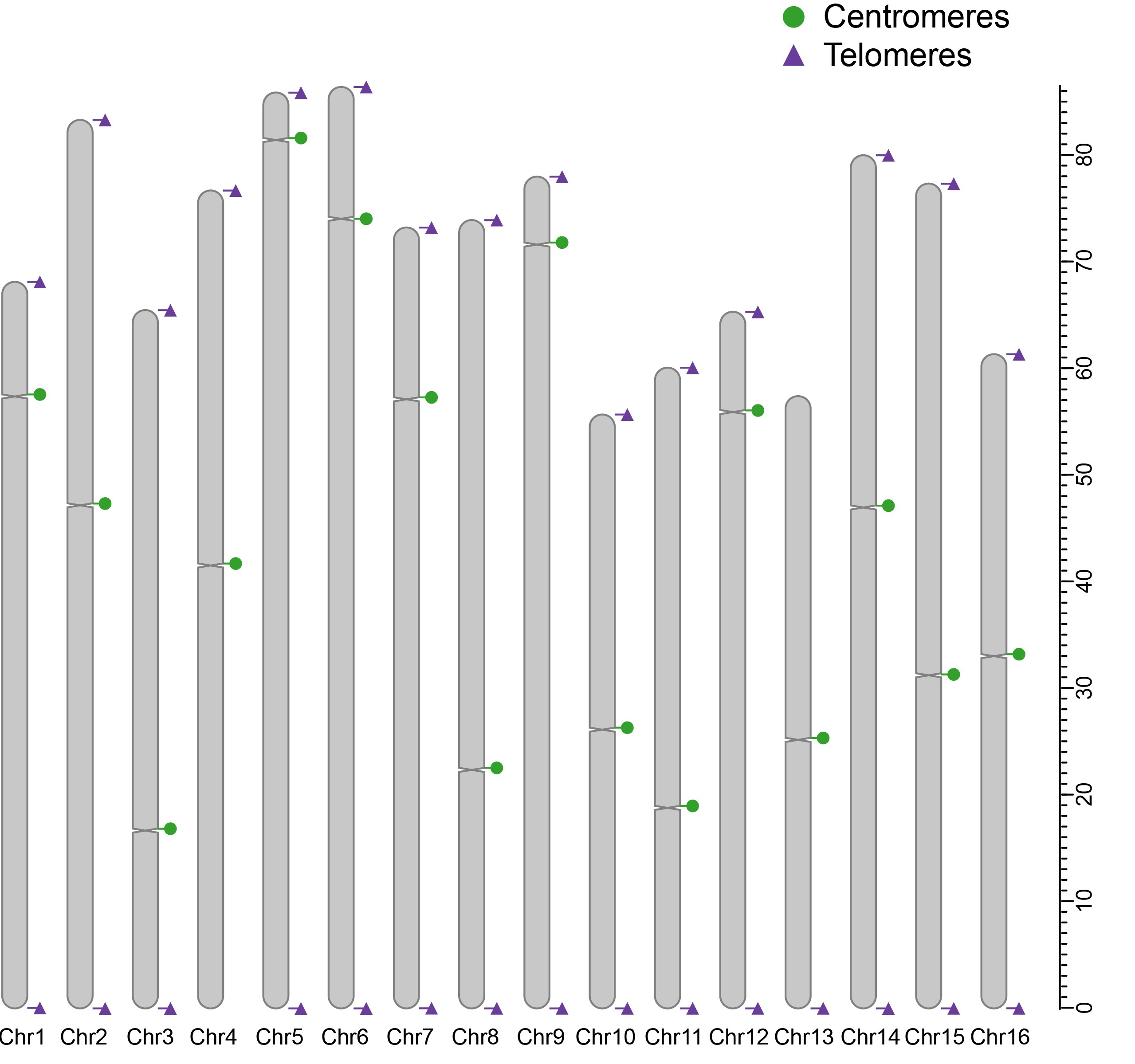

Supplement: Supplementary file 3 — Supplementary Material 3. Figure S3. The locations of centromeres and telomeres on each chromosome in the chimeric genome. [file 43897_2025_192_MOESM3_ESM.jpg]

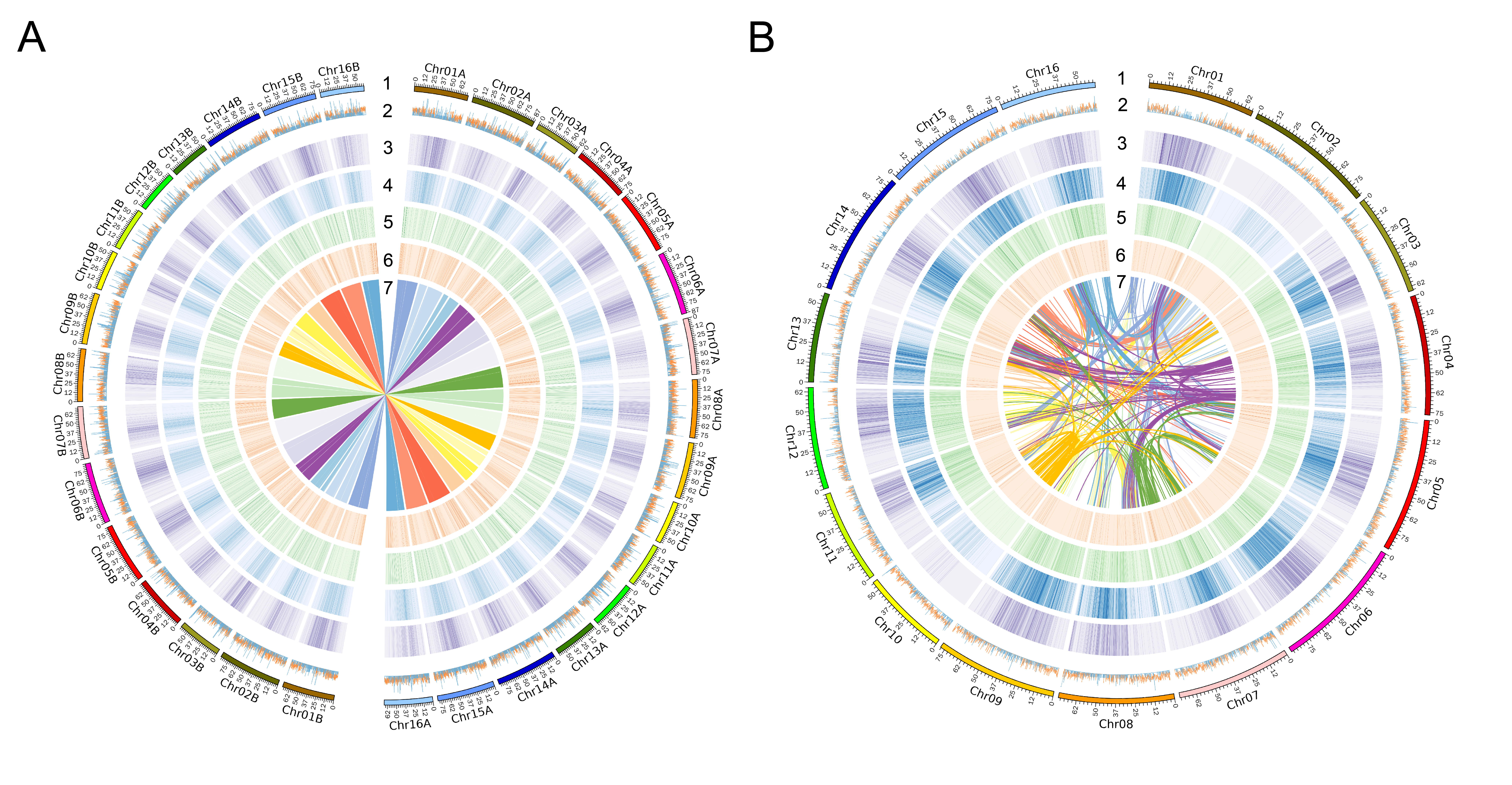

Supplement: Supplementary file 4 — Supplementary Material 4. Figure S4. Circos plot of Z. elliottiana genomes. A The left panel shows the haplotype-resolved genome. B The right panel shows the chimeric genome. (a) Chromosome length and number. (b) Orange and blue lines represent gene density and noncoding RNA density, respectively. (c) Density of Copia-type LTR-RTs. (d) Density of Gypsy-type LTR-RTs. (e) DNA transposon density. (f) Gene expression levels. (g) syntenic blocks. [file 43897_2025_192_MOESM4_ESM.jpg]

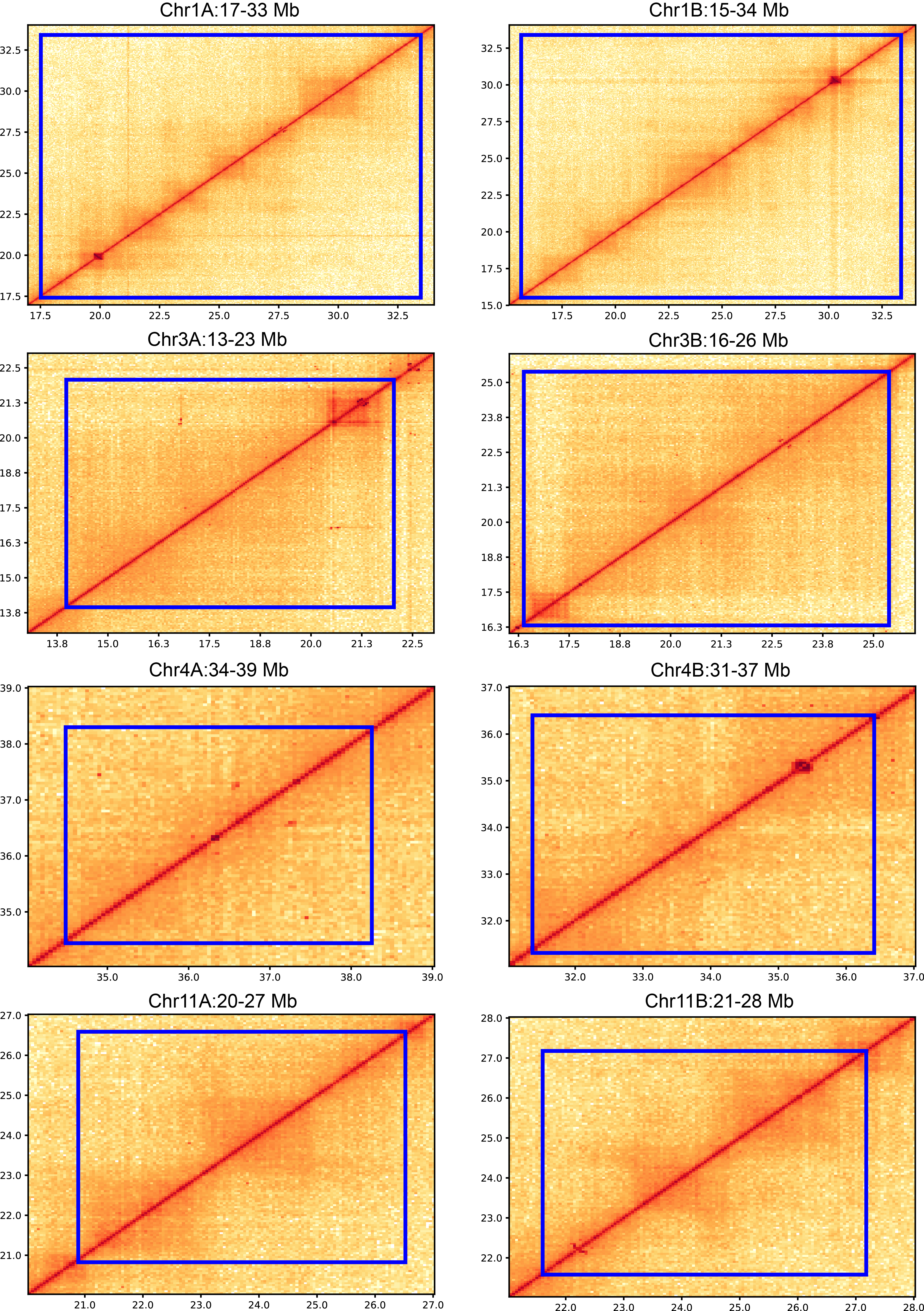

Supplement: Supplementary file 5 — Supplementary Material 5. Figure S5. Local Hi-C heatmaps were used to validate the accuracy of genomic variation detection between homologous chromosomes. [file 43897_2025_192_MOESM5_ESM.jpg]

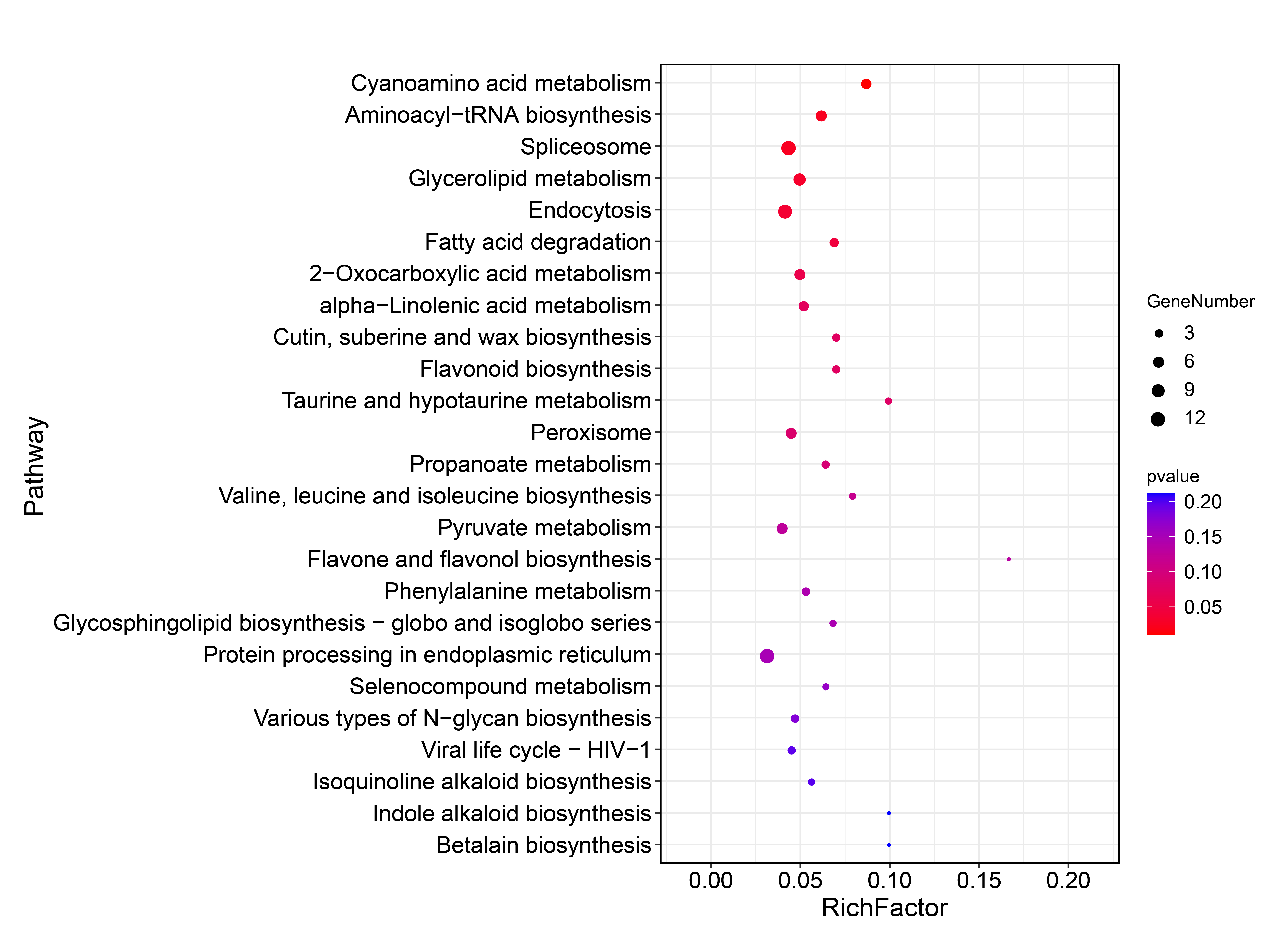

Supplement: Supplementary file 6 — Supplementary Material 6. Figure S6. KEGG enrichment analysis of genes affected by genomic variation between the two haplotypes of Z. elliottiana genome. [file 43897_2025_192_MOESM6_ESM.jpg]

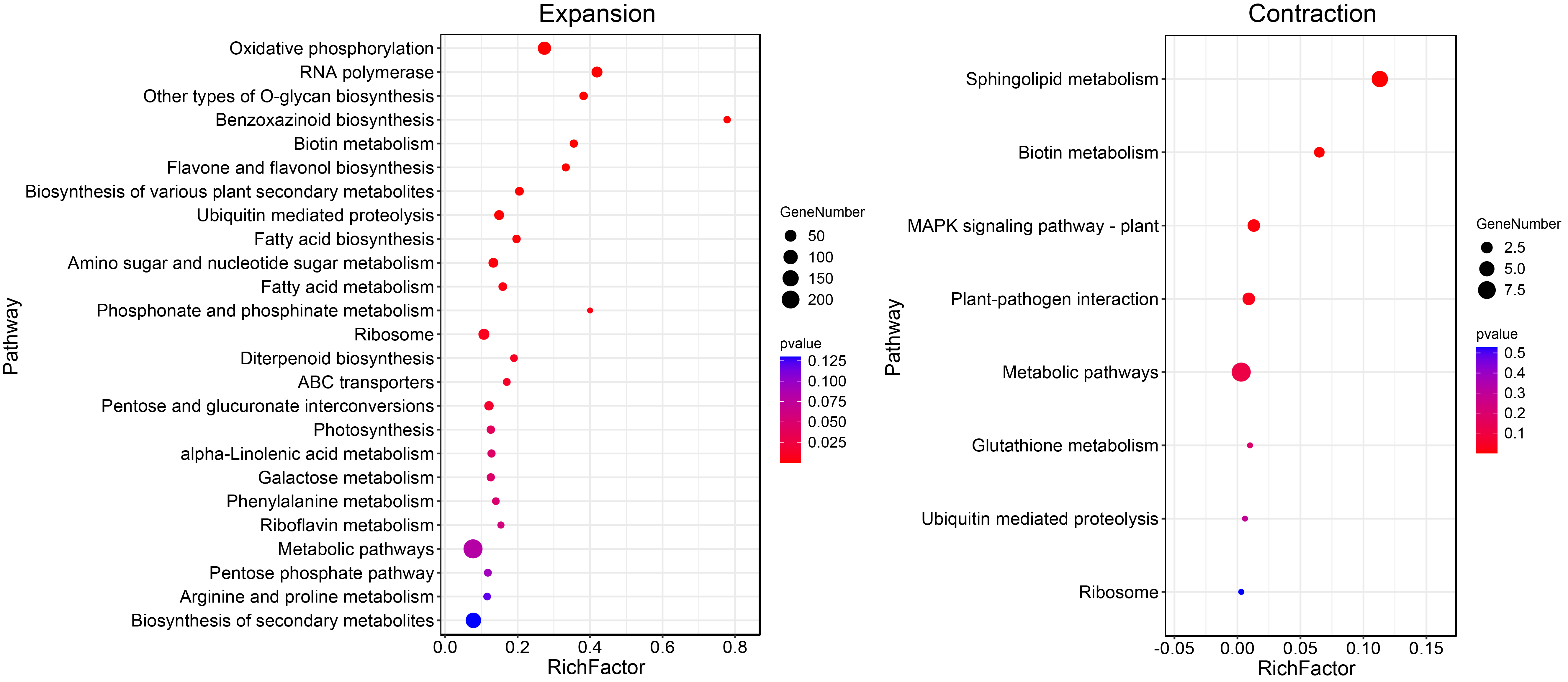

Supplement: Supplementary file 7 — Supplementary Material 7. Figure S7. KEGG enrichment analysis of expanded/contracted gene families in Z. elliottiana. [file 43897_2025_192_MOESM7_ESM.jpg]

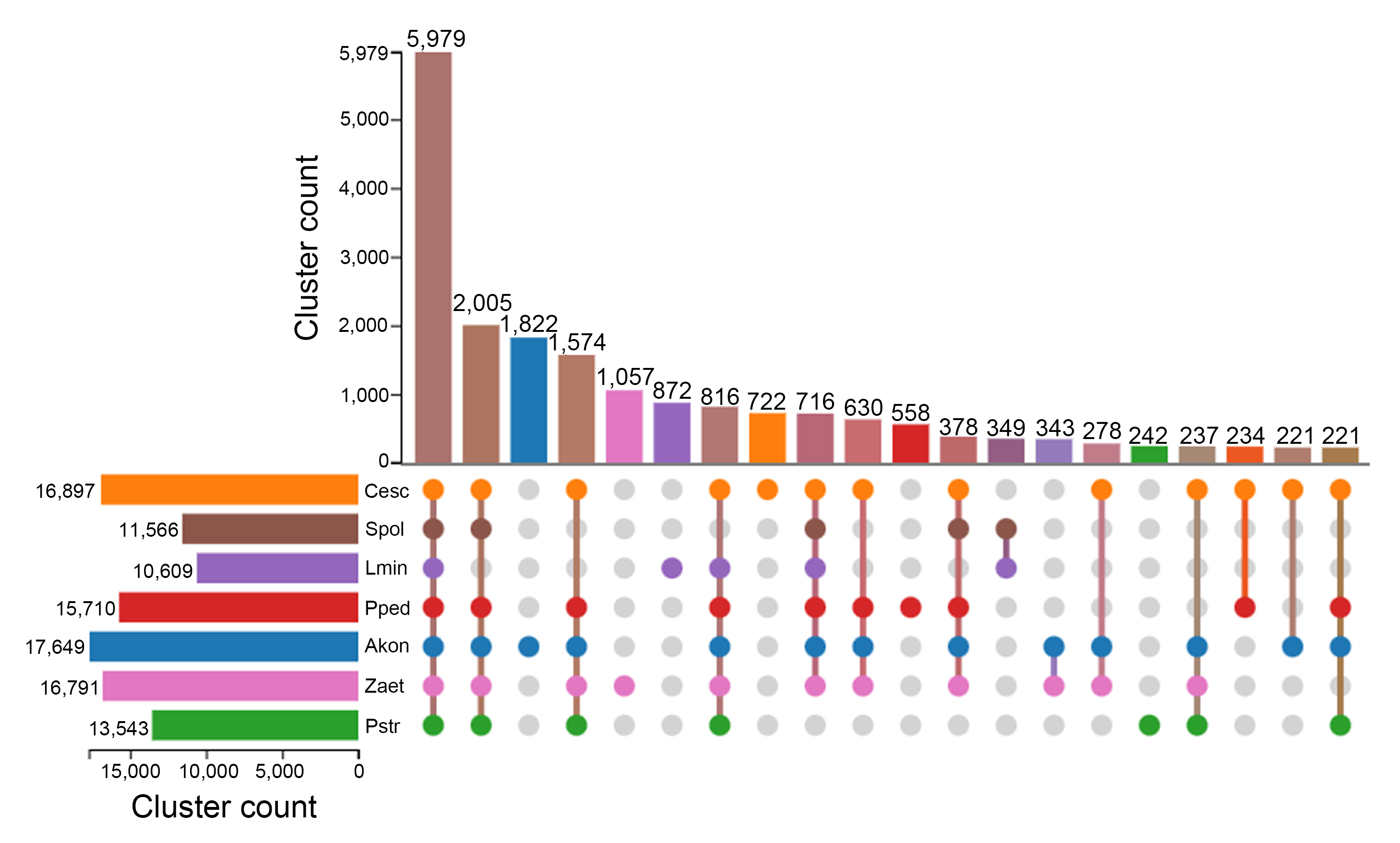

Supplement: Supplementary file 8 — Supplementary Material 8. Figure S8. Gene family clustering analysis of 7 Araceae species. The species included A. konjac, C. esculenta, P. pedatisecta, P. stratiotes, Z. elliottiana, S. polyrhiza, and L. minuta. [file 43897_2025_192_MOESM8_ESM.jpg]

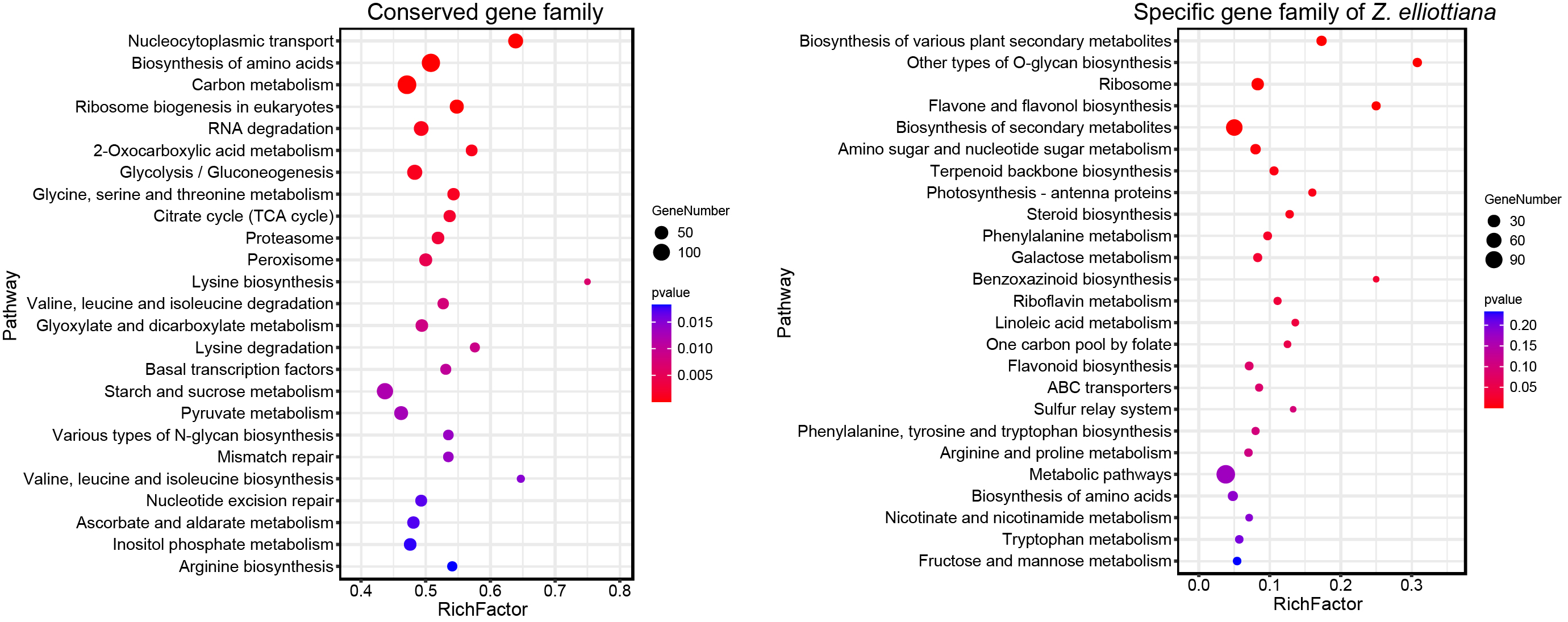

Supplement: Supplementary file 9 — Supplementary Material 9. Figure S9. KEGG enrichment analysis of conserved gene families in Araceae and specific gene families in Z. elliottiana from gene family cluster analysis. [file 43897_2025_192_MOESM9_ESM.jpg]

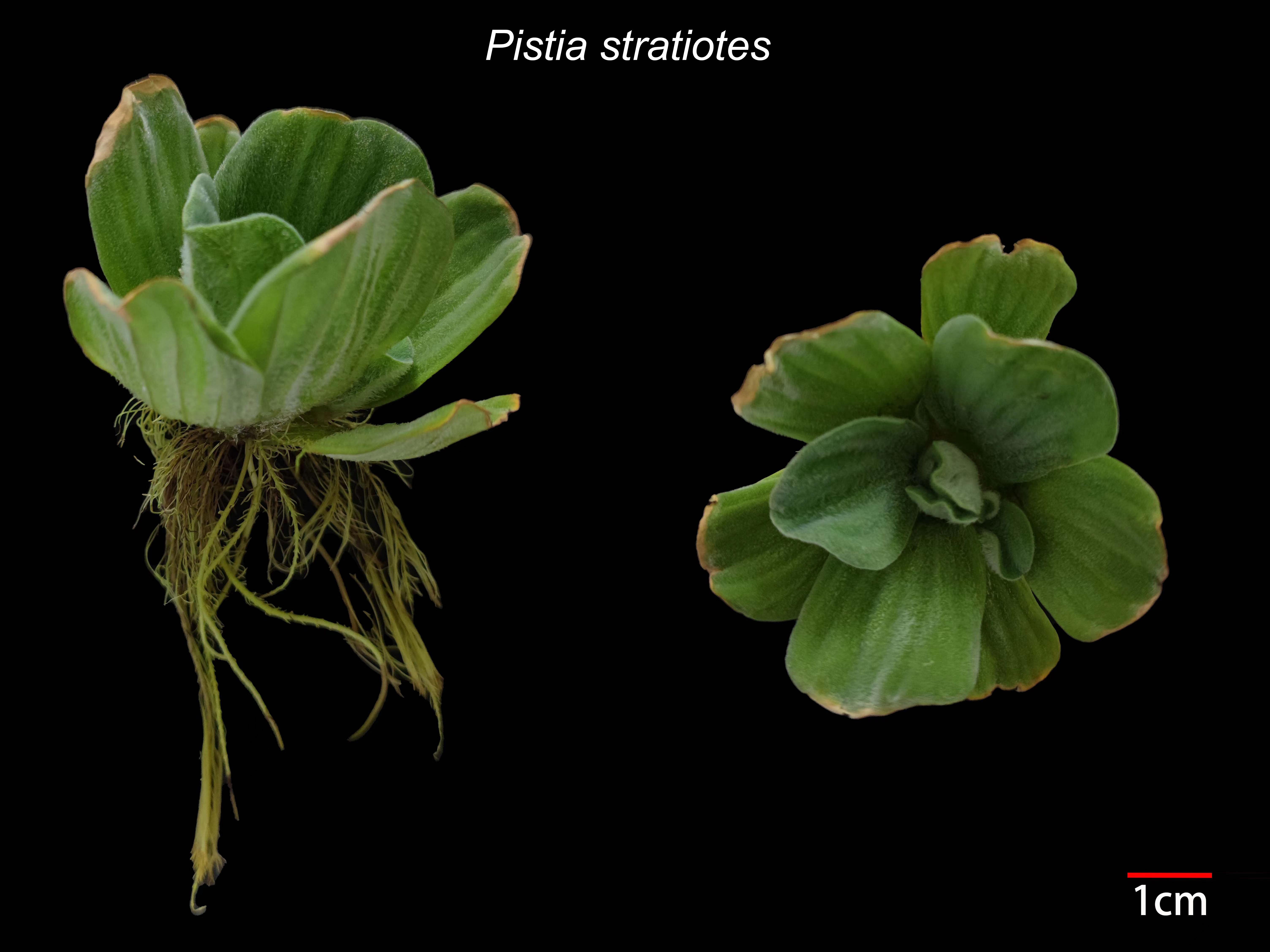

Supplement: Supplementary file 10 — Supplementary Material 10. Figure S10. Photographs of P. stratiotes used to reveal floating aquatic plants in the True Araceae family. [file 43897_2025_192_MOESM10_ESM.jpg]
